# Disentangling the Taxonomy, Systematics, and Life History of the Spider-Parasitic Fungus *Gibellula* (Cordycipitaceae, Hypocreales)

**DOI:** 10.3390/jof9040457

**Published:** 2023-04-08

**Authors:** Thairine Mendes-Pereira, João Paulo Machado de Araújo, Thiago Gechel Kloss, Diogo Henrique Costa-Rezende, Daniel Santana de Carvalho, Aristóteles Góes-Neto

**Affiliations:** 1 Laboratory of Molecular and Computational Biology of Fungi, Department of Microbiology, Instituto de Ciências Biológicas, Universidade Federal de Minas Gerais, Belo Horizonte 31270-901, MG, Brazil; thairinemp@gmail.com; 2 Institute of Systematic Botany, The New York Botanical Garden, 2900 Southern Blvd., Bronx, NY 10458-5126, USA; 3 Laboratory of Behavioral Ecology, Department of General Biology, Universidade Federal de Viçosa, Viçosa 36570-900, MG, Brazil; 4 Department of Biological Sciences, Universidade Estadual de Feira de Santana, Feira de Santana 44036-900, BA, Brazil; 5 Laboratory of Mycology, Department of Microbiology, Instituto de Ciências Biológicas, Universidade Federal de Minas Gerais, Belo Horizonte 31270-091, MG, Brazil; 6 Graduate Program in Bioinformatics, Universidade Federal de Minas Gerais, Belo Horizonte 31270-901, MG, Brazil

**Keywords:** host–parasite interaction, arthropod pathogen, *Gibellula*, Ascomycota, molecular phylogeny

## Abstract

*Gibellula* (Cordycipitaceae, Hypocreales) is frequently observed growing on spiders, but little is known about their host range. One of the greatest challenges in describing these interactions is identifying the host, since the fungus often rapidly consumes the parasitised spiders and destroys important diagnostic taxonomic traits. Additionally, the global diversity of *Gibellula* remains unclear, as does the natural history and phylogenetic relationships of most of the species. Herein, we performed an extensive investigation on the species of *Gibellula*, reconstructed the most complete molecular phylogeny of the genus in the context of Cordycipitaceae, and performed a systematic review in order to provide the foundations towards a better understanding of the genus. Therefore, we have performed an integrative study to investigate the life history of the genus and to disentangle the questionable number of valid species proposed over time. We provided novel molecular data for published species that had not been sequenced before, such as *G. mirabilis* and *G. mainsii*, and evaluated all the original and modern morphological descriptions. In addition, we presented its global known distribution and compiled all available molecular data. We suggested a set of terms and morphological traits that should be considered in future descriptions of the genus and that a total of 31 species should be considered as accepted.

## 1. Introduction

Several entomopathogenic fungi induce changes in host arthropods, which may optimise fungal dispersion. One important route of transmission involves parasitic fungi that dislodge hosts to die suspended from specific places [1,2,3]. This strategy is usually employed by fungi that grow outside the host body after infection to reach a next suitable host [4], such as those inducing infected ants to die suspended while attached to vegetation [1,2,5]. These parasites generally induce host death in a specific place of the vegetation to increase their fitness, development, and transmission [1,6,7].

Host manipulations that result in host displacement are relatively well-documented strategies of transmission employed by some lineages of entomopathogenic fungi in the order Hypocreales [8]. Those behaviour-manipulating lineages affect distinct arthropod groups, mostly insects, such as ants [1,9], wasps [10], beetles [11], locusts [3], flies [3,12], cicadas [13], and moth or butterfly caterpillars [11,12]. However, recent studies have suggested that spiders can also be parasitised and displaced to a different habitat by *Gibellula* species [14,15]. For some hosts (e.g., ants and wasps), host identification based on morphological features is usually achievable [9]. Nonetheless, in the case of arthropods such as spiders, the parasitic fungi are commonly encountered completely covering the host, often destroying important diagnostic taxonomic traits critical for host identification, as is commonly observed in spiders colonized/infected by *Gibellula* species. Therefore, imprecise records of host identification usually underestimate the diversity of infected hosts and, consequently, the potential effects on their behaviour caused by the fungi [5,16].

The infection of spiders by *Gibellula* species is probably globally distributed [17,18,19,20]. However, records have been often reported as simple taxonomic notes, which do not include information on the ecology of the parasite or explore the genetic information to determine its phylogenetic placement within Cordycipitaceae. When infected by *Gibellula* species, different families of spiders are found dead on the abaxial face of leaves [5,12,15,18], where some of them do not naturally occur [21]. Although *Gibellula* spp. are widely described as specific parasites of spiders [17], aspects of their parasitism strategy are unknown, such as whether species in the genus parasitise specific hosts, as is the case with other fungi that have similar phenotypes (i.e., the ones which induce the host to die suspended). Elucidating the evolutionary advantage of these interactions can improve our understanding of how *Gibellula* parasites become established in a certain host, persist in the environment, and have radiated in evolutionary time.

The genus *Gibellula* is classified as a member of Cordycipitaceae (Hypocreales, Ascomycota) [22]. Species within this genus form one or multiple synnemata, which are compact and erect conidiomata that harbour *Aspergillus*-like or, less commonly, *Penicillium*-like conidiophores with terminal vesicles. These vesicles bear hyaline metulae, phialides, and conidia and, usually, do not produce sexual structures. The presence of sexual structures embedded in the subiculum in some species, such as *Gibellula arachnophila*, *G. aranearum*, *G. clavata*, *G. clavulifera* var. *alba*, *G. dabieshanensis*, *G. dimorpha*, *G. leiopus*, and *G. pulchra* has led to an erroneous description of the sexual form of these species as a different genus, *Torrubiella* [19,20,23,24].

The species within *Torrubiella* have traditionally been used to classify pathogens of arthropods (mostly infecting spiders but also scale insects) that produce superficial perithecia on a loose mycelial mat. This mat is known as a subiculum and is formed directly on the hosts (rather than erected on a stalk) [23]. Based on their morphology, ecology, and molecular studies, *Torrubiella* was considered as the teleomorph of *Gibellula* and other genera [16,25]. The recommendation of the International Code of Nomenclature for algae, fungi, and plants [26] is to combine the terms proposed for teleomorphs and anamorphs into one representative name, known as 1F1N (one fungus one name). In addition, the molecular phylogenetic investigation of Cordycipitaceae, as well as morphological comparisons [27], provided the foundation to maintain the name *Gibellula* rather than *Torrubiella* for both asexual and sexual morphs.

The type species of *Gibellula* is *G. pulchra* Cavara [28], originally described as *Corethropsis pulchra.* Currently, the genus *Gibellula* contains 54 records of species names at Index Fungorum (www.indexfungorum.org, accessed on 1 November 2022) [29] and 58 records at Mycobank (https://www.mycobank.org, accessed on 1 November 2022) [30]. In the latter case, the number of species is controversial and will be discussed herein. *Gibellula* taxonomy is broadly based on morphological studies [19,23,31], and relatively few species have multiloci data and have been included in phylogenetic studies [15,18]. Furthermore, the natural phenotypic variation across specimens and multiple cases of synonyms have led to some conflicts in its taxonomy. For instance, 11 species were synonymized under *G. pulchra* and 5 species under *G. leiopus*, which are the most commonly found species of *Gibellula* [19,20,27,32], and a summary of this is given by [31]. Conversely, there is no consensus about the taxonomic and nomenclatural acceptance of *Gibellula petchii* (see [18,20]).

Newly proposed species of *Gibellula* have included both morphological and molecular characters. Although it is enough to support the hypothesis of these records as new species, the molecular representativeness of the species of *Gibellula* is still low. Of the 54 putative species, only 18 species are included in phylogenetic studies [15,18,33,34,35,36]. Because of this, most phylogenies were built for specific systematic purposes (e.g., describing new taxa) without providing an overview of the genus.

Considering the difficulties in using morphological characters to delimit *Gibellula* species and their hosts [16], and the lack of DNA sequence information for several species, most of them being based on old and scarce type materials, a thorough data compilation and standardisation of the *Gibellula* species would be very useful for providing a framework for the classification and ecology of the genus. Thus, we performed a comprehensive worldwide systematic review of *Gibellula* species. For this, we assessed the taxonomic fungal characters relevant for host–parasite interactions; revised all described species and provided a polyphasic taxonomic reappraisal; documented their global geographic distribution; and constructed the first host–parasite interaction network. Additionally, we conducted a phylogenetic study of the genus *Gibellula* and related taxa from Cordycipitaceae, including novel molecular data from four representative species of *Gibellula* collected in the Atlantic Rainforest: *G. pulchra*, *G. leiopus*, *G. mirabilis*, and *G. mainsii*. This study represents the first integrative treatment of the genus in which morphological, phylogenetic, and host range data are combined to investigate the life history and the evolution of the genus *Gibellula*.

## 2. Methods

### 2.1. Systematic Review Approach

#### 2.1.1. Eligibility Criteria and Information Sources

We performed a comprehensive review of the literature following the Preferred Reporting Items for Systematic Reviews and Meta-Analyses (PRISMA) 2020 protocol (Figure S1) [37]. We searched for information that indicates the study is related to fungal parasites of spiders, and the eligibility criteria for inclusion of studies consisted of considering papers that present records of interaction between entomopathogenic fungi and spiders, *Gibellula* sp. morphological descriptions, and geographic distribution.

We conducted the literature searches from July to November 2022, in three databases: *Web of Science–Core Collection, Scopus* (Elsevier, Amsterdam, The Netherlands), and *JSTOR*. Searches were performed without limitation of date. We complemented our search by scanning the reference lists of the papers that fulfilled our inclusion criteria and checking whether all the species of the genus and synonyms recorded at Index Fungorum were included.

#### 2.1.2. Search Strategy, Study Selection, and Data Collection

We conducted the searches using each keyword separately, for the following terms: ‘arachnid* AND fung*’; ‘Ascomycota AND spider’; ‘Cordycipitaceae AND spider’; ‘entomogen* AND spider’; ‘entomopathogen* AND spider’; ‘*Granulomanus* AND spider’; ‘Hypocreales AND spider’; ‘spider AND pathogen* AND fung*’; ‘*Gibellula*’. For each record identified through database searching, we evaluated the title, abstract, and keywords. For studies that provided information regarding fungal parasites and spiders, we sought the full text, looking for primary studies about the taxa of interest (i.e., *Gibellula*, some species of *Torrubiella* that were considered teleomorphs of *Gibellula*, or synonyms) written in English, and which provided a fungal description and accurate records.

For each paper selected, we investigated the methods section for precise records of fungal collections and hosts, if available. We also recorded each trait from the fungal morphological description, considering fungal traits and molecular data (when available) of each species. For the studies that described more than one species, we included all the records in the review. For some species, the only record was published in a different language (e.g., in Russian, Italian, and Latin); therefore, we translated the description to English and included the relevant information. The studies that reported the occurrence and distribution of the species without fungal description were evaluated and included in the global distribution analyses, only if they reported a new location described for the fungus that was not detected in an original paper with a description and was not duplicated in reviews (n = 10). After applying our inclusion criteria, a total of 68 studies were included from the search on databases and 31 from reference lists.

#### 2.1.3. Data Items and Summary Measures

We divided the data into four main sets to perform the analyses: morphological characters, molecular data, geographic distribution, and host diversity. The description of morphological characters consisted of traits from descriptions of micro- and macroscopic characters. We considered those characters that were described for more than 70% of the species. Categorical variables consisted of characters such as the presence of unique traits of the species, shape of synnemata, colour of mycelia, and shape of conidiophores, vesicles, metulae, phialides, and conidia. When more than one state was described for the same character, we selected the most representative or most frequent. Subsequently, we attributed a code for each character to create a numerical matrix. Continuous variables consisted of fungal traits, such as size of conidiophores, conidial heads, metulae, phialides, and conidia. As all the measures showed a large variance, we used the minimum and maximum values of each character for the analysis.

### 2.2. Fieldwork Areas and Collection of Under-Represented Species

We collected dead spiders parasitised with *Gibellula* spp. from January 2020 to January 2022 to improve the molecular dataset for under-represented species. Fieldworks were performed at three conservation units of the Atlantic Rainforest, located in the southeastern region of Brazil: Parque Estadual do Rio Doce (Marliéria, MG, 19°45′45″ S, 42°37′19″ W); Estação Biológica de Santa Lúcia (Santa Teresa, ES, 19°57′56″ S, 40°32′25″ W); and Reserva Biológica Augusto Ruschi (Santa Teresa, ES, 19°54′45″ S, 40°33′11″ W). These localities were selected according to previous observations of spider–*Gibellula* interactions. Fungi were carefully collected along the host spiders and substrates, as described in [15]. Fungal and spider specimens were collected under permission of the Instituto Chico Mendes de Conservação da Biodiversidade (ICMBio-68171), and specimens were deposited at the Centro de Coleções Taxonômicas (CCT-UFMG) (https://www2.icb.ufmg.br/cct/, accessed on 1 January 2022) [38], Institute of Biological Sciences, Universidade Federal de Minas Gerais.

### 2.3. DNA Extraction, Amplification, and Sequencing

We extracted the genomic DNA directly from the specimens collected. From the species that had aerial synnemata, we removed them from the parasitised spiders and macerated only the fungus in a microtube in contact with liquid nitrogen. The specimens that did not have aerial structures were macerated along the spider hosts. Genomic DNA was extracted using the ZymoBIOMICS DNA Miniprep kit (Zymo Research^®^, Irvine, CA, USA), according to the manufacturer’s protocol. The quality of DNA was assessed with agarose 1% gel electrophoresis, and DNA concentration was measured by spectrophotometry in NanoDrop^®^ (Thermo Scientific^®^, Waltham, MA, USA). We amplified six genomic regions: (i) small (SSU) and (ii) large (LSU) nuclear ribosomal subunits, (iii) Internal Transcribed Spacer (ITS), (iv) translation elongation factor 1-α (TEF), (v) RNA polymerase II largest subunit (RPB1), and (vi) RNA polymerase II second largest subunit (RPB2). Primers and conditions used to amplify each region are described in Tables S1 and S2, respectively [39,40,41,42,43,44]. Polymerase chain reactions (PCRs) were performed in a final volume of 25 µL and followed the methods described in Mendes-Pereira et al. [15]. Sequences (forward and reverse) were edited and assembled into contigs in Geneious Prime^®^ 2022.1.1 [45]. All sequences of *Gibellula* sp. were compared using BLASTn and their morphological data were compared to descriptions from literature to confirm their generic identity.

### 2.4. Analyses of Morphological Traits

Matrices of morphological data from databases were concatenated into a single dataset. We included 43 species descriptions, with 11 categorical and 9 continuous variables correlated to fungal traits. The dataset presented approximately 15% of missing data. We performed a Non-Metric Dimensional Scaling (nMDS), considering the Bray–Curtis coefficient of similarity [46]. Analyses were performed in Past. v. 4.08 [47].

Morphological data from the representative specimens collected in this study were obtained by examining synnemata and conidiophores on slides mounted with water, KOH 3%, and eosin or lactophenol and cotton-blue for light microscopy. All slides were photographed using an Olympus BX50 microscope and used to compare the micromorphological structures from our specimens to the original descriptions of *Gibellula* spp. (Table S3). Moreover, we inspected the type specimens deposited at Kew Garden Fungal Collection (HerbIMI) of the species *G. alata* (Herb IMI 339724), *G. eximia*, and *Torrubiella gibellulae* (*G. aranearum* R 404).

### 2.5. Multigene Phylogenetic Analyses

#### 2.5.1. Obtaining Molecular Data

Sequences previously deposited in the NCBI-GenBank database as *Gibellula* were checked and included in our phylogenetic analyses, removing those that were too divergent to avoid misinterpretation in the global analyses (e.g., *Gibellula curvispora* JQ342826 and *Gibellula formosana* MT924519, both without similarity with *Gibellula* sp.), as well as 25 strains that were previously identified only at the genus level. Sequences from Cordycipitaceae from 21 genera that are listed in Genbank Taxonomy were also included. Additionally, we generated sequences from 14 representative specimens, comprising four published species (Table S4).

#### 2.5.2. Phylogenetic Reconstruction

We used a multigene approach to construct the phylogeny for *Gibellula* to place the genus within the Cordycipitaceae family. The alignments of each genomic region were performed using Geneious Prime 2022.1.1 with the MUSCLE algorithm, checked for ambiguity among the nucleotides, and gaps were considered as missing data. We concatenated the six genomic regions into a single combined dataset using Geneious Prime 2022.1.1. After sequence selection, the final alignment length was 6197 bp (SSU: 1097, ITS: 704, LSU: 1344, RPB1: 826, RPB2: 1161, TEF: 1065 bp) and consisted of 307 taxa.

For selecting evolutionary models for phylogenetic analyses and data partition, we used ModelFinder [48,49]. The dataset consisted of five data partitions, based on Bayesian Information Criterion (BIC) scores (Table S5). We used the same partition definitions to reconstruct our phylogenetic trees using both Maximum Likelihood and Bayesian Inference analyses. Maximum Likelihood analyses were performed with IQ-TREE multicore v. 1.6.12 [50,51] for ITS, the region used as a barcode for most of the fungal species and that presents the most complete dataset available (Figure S2). We then performed the analysis again using the concatenated dataset containing the six genomic regions. We employed the nucleotide substitution models and partitions selected using ModelFinder during the generation of 1000 bootstrap replicates for both analyses.

Bayesian Inference analyses were performed with the BEAST2 v. 2.7.3 [52] using the CIPRES Science Gateway v.3.3 [53]. A parallel run, consisting of four chains, was subjected to Markov Chain Monte Carlo (MCMC) analyses until the runs converged with a split frequency < 0.01. The MCMC analysis started with a heating parameter of 0.1 from a random tree topology. The concatenated analysis lasted 30 million generations, and trees were saved every 1000 generations. Finally, 25% of trees were discarded as the burn-in phase, and the remaining trees were used to build a maximum clade credibility tree using TreeAnnotator v.2.6.6 [52], and phylogenetic trees were viewed using FigTree v. 3.5.9 [54].

We compared the tree topologies, and the numerical values on branches indicate bootstrap percentages (ML > 50%) obtained by the Maximum Likelihood analysis. The scale bar on the bottom shows nucleotide substitutions per site. Trees were edited using Inkscape (www.inkscape.org, accessed on 1 December 2022) [55], and the species *Ophiocordyceps gracilis* EFCC 8572 (Ophiocordycipitaceae) was used as an outgroup to root the trees.

### 2.6. Global Distribution

In order to investigate the geographic distribution of *Gibellula* sp., we extracted the collection locality of each specimen from databases and from fieldwork and obtained their coordinates from the papers or from Google Earth Pro v. 7.3.3.7786 (http://www.google.com/earth/index.html, accessed on 1 November 2022) [56]. We then converted the coordinates to decimal degrees using the Convert Geographic Units, provided by Montana State University (http://rcn.montana.edu/Resources/Converter.aspx, accessed on 1 November 2022) [57], and created a global map showing the species occurring in each country using the packages *ggplot2* v. 3.3.3 [58] and *scatterpie* v. 0.1.8 in R software [59]. Locations reported in reviews or collections were evaluated to avoid repetitions.

### 2.7. Host–Parasite Interaction

Considering that *Gibellula* sp. is described as a spider-specific genus [16], and earlier descriptions in different hosts were reviewed [20,60], we used the host description as one of the criteria to state whether a species should be maintained or excluded from the genus. We then connected the species of *Gibellula* (n = 21) and families of spiders (n = 15) that were reported as hosts. The presence/absence matrix was converted to a bipartite network where parasite and host groups represent two sets of nodes. The edges between the nodes of distinct sets were created when the *Gibellula* species parasitised a spider family. The node size is proportional to the node degree: nodes (species/family) with a higher number of connections are proportionally larger than the ones with fewer connections. The network was generated using the software GePhi v. 0.9.2 [61].

## 3. Results

### 3.1. Systematic Review Approach

The database search identified 4381 records for the 9 keywords, which were then filtered to 210 records after removing the records in which the main subjects were not related to *Gibellula*. We screened 210 papers from databases and 31 additional records identified through other sources (citation searching). After duplication removal (n = 142), we removed two studies that did not provide access to the full text. Among the remaining full-text articles (n = 97), 42 studies were excluded (i) for describing different taxa (n = 26), (ii) for not providing a fungal description or showing inaccurate records (n = 9), and (iii) for providing repetitive information or not primary research (n = 6). The analyses were then performed with 56 selected articles (Figure S1).

### 3.2. Indexation, Validity, and Currently Accepted Species

Considering the former names attributed to the asexual (*Gibellula*) and sexual (*Torrubiella*) morphs, we found 56 names in our database. Nonetheless, 54 names are recorded at Index Fungorum and 58 at Mycobank. When we checked the validity of these names at Mycobank [30], 50 names were considered legitimate. One species is not registered at Mycobank: *Gibellula formosana* (Index Fungorum ID 646527, Sawada 1919; unfortunately, this paper is not available). Moreover, two species are considered as invalid names, according to Art. 36.1 of the International Code of Nomenclature for Algae, Fungi, and Plants [26]: *Gibellula araneicola* and *Gibellula tropicalis*.

**Figure 1:**
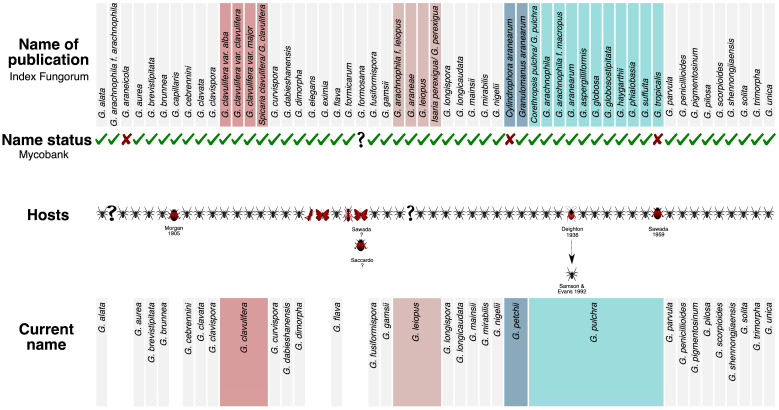
Currently accepted species of the genus *Gibellula* and associated hosts. Names of publications for the genus were verified at Index Fungorum (top), as well as their synonyms. The name status was confirmed at Mycobank and considered legitimate (indicated by a green symbol √), invalid/illegitimate (red symbol ×), or not registered (black symbol ?). Hosts described in the papers are indicated by spiders (black); non-spider hosts (red) from the orders Orthoptera, Lepidoptera, Hymenoptera, Hemiptera (later revised), and other insects (illustrated as Coleoptera) were further removed from our analyses. Current names according to the databases (bottom) are shown as the following: grey squares: species that did not display taxonomic conflicts; dark brown squares: different variations proposed for *Gibellula clavulifera* (n = 4); light brown squares: species synonymised as *Gibellula leiopus* (n = 5); dark blue squares: *Gibellula petchii*, suggested to comprise *Cylindrophora aranearum* and *Granulomanus aranearum*, but still under evaluation; light blue squares: names synonymised as *Gibellula pulchra* (n = 11), comprising 31 species remaining as accepted names.

Based on the literature and the database, 26 species (out of the 50 legitimate) did not display taxonomic conflicts (not under a synonym) (Figure 1, grey squares). *Gibellula arachnophila f. leiopus*, *G. araneae*, and *G. perexigua* were considered synonyms of *G. leiopus* by more than one review [19,24,31,62]. Humber and Rombach [63] suggested the species *G. petchii* to comprise *C. aranearum* (formerly described as the asexual morph of *Torrubiella albolanata*) and the synonym *Granulomanus aranearum* (dark blue square), but this is still questioned [20,31]. *Gibellula pulchra* was firstly attributed as the current name of *C. pulchra* [28] and, later, as the synonym of *G. arachnophila*, *G. arachnophila f. macropus*, *G. aranearum*, *G. aspergilliformis*, *G. globosa*, *G. globosostipitata*, *G. haygarthii*, *G. phialobasia*, *G. suffulta,* and *G. tropicalis* [19,20,24,27,31,32].

### 3.3. Similarities among Morphological Traits

The Non-Metric Dimensional Scaling (nMDS) performed with morphological descriptions for the genus *Gibellula* revealed similarities reported in different reviews, but with a poor value of similarity (S= 0.2029, Figure 2). Species descriptions available for three different forms of *G. clavulifera* are in opposite edges (purple). Moreover, the Minimum Spanning Tree (MST) indicated that most of the species synonymised as *G. pulchra* (blue) are not closely related, as well as the two descriptions of *G. leiopus* (green).

After comparing the micromorphological structures from our specimens to the original descriptions of *Gibellula* spp., we detected that we had added molecular and morphological information for species that were previously under-represented in phylogenies: *G. leiopus*, *G. pulchra*, *G. mainsii,* and *G. mirabilis* (Figure 3; Table S3).

### 3.4. Multigene Phylogenetic Analyses

Sequences of 302 specimens from Cordycipitaceae, including 84 of *Gibellula*, were used for the phylogenetic analyses (Table S4). Amongst the 54 species formerly described as *Gibellula*, the 22 known species with available data at molecular databases clustered in a clade (BS = 96%) splitting into 9 distinct sub-clades: (i) *G. pulchra* complex: *G. pulchra*, *G. brevistipitata*, *G. nigelii*, *G. flava*, *G. pilosa*, *G. unica*, and *G. solita*; (ii) *G. mirabilis*; (iii) *G. longispora*/*G. parvula*; (iv) *G. pigmentosinum*; (v) *G. gamsii*; (vi) *G. aurea*; (vii) *G. trimorpha*; (viii) *G. cebrennini*/*G. fusiformispora*/*G. longicaudata*; (ix) *G. penicillioides*/*G. scorpiodes; G. leiopus* is split into three different clades. Furthermore, specimens of *G. clavulifera* did not group in the same clade either. The position of *G. clavulifera* var. *alba* (ARSEF1915) was also distant from the other isolates of the species. Among the other Cordycipitaceae genera, *Lecanicillium, Cordyceps*, and *Simplicillium* are polyphyletic, presenting multiple origins. In addition, two specimens of *Jenniferia thomisidarum* are positioned inside the clade of *Gibellula*, but these specimens only have two genomic regions available, which may reflect incorrect placement (Figure 4 and Figure S3).

Some isolates described as *Torrubiella arachnophila/T. arachnophilus* grouped in two different clades. As the name *T. arachnophila* was attributed to more than one species in the literature (e.g., *G. pulchra* and *G. leiopus*), we identified isolates that can group with *G. pulchra* (*T. arachnophilus* BUG507) and *G. leiopus* (*T. arachnophilus 1*), and can potentially be attributed to these species. Therefore, based on morphological descriptions and molecular data, we suggest that, currently, 31 species remain as accepted names for the genus *Gibellula*.

### 3.5. Global Distribution

Species of *Gibellula* infecting spiders have been recorded in 33 countries. A total of 14 species are reported in a single place, while *G. leiopus* and *G. pulchra* are distributed worldwide, found in 20 and 45 localities, respectively. The latitudinal distribution of *Gibellula* ranged from 31° S (Chile) to 61° N (Russia), while the longitude varied from 155° W (Waimea, HI, USA) to 160° E (Guadalcanal, Solomon Islands). We attributed the names we considered as currently accepted species in this review and showed their global distribution (Figure 5).

### 3.6. Host–Parasite Interaction

As host specificity is considered a criterion of exclusion of the genus [16], we highlighted the species described in non-spider hosts. Different hosts were described for *Gibellula capillaris* (small insect) [64], *Gibellula elegans* (grasshopper) [65], *Gibellula eximia* (butterfly) [66], and *G. formicarum*, *G. formosana* Sawada, and *G. tropicalis* (other insects) [67] (Figure 1). *Gibellula formicarum* was mistakenly described as parasitising ant hosts and further transferred to *Pseudogibellula* as *P. formicarum* based on the conidial ontogeny [60]. *Gibellula aranearum* had been formerly described as parasitising Hemiptera [68], but the host was posteriorly re-evaluated by Samson and Evans [20] as a spider. We checked the type specimen deposited at the Kew Garden Fungal Collection as *G. eximia* and, other than the host clearly being another arthropod, the fungus did not resemble *Gibellula.* This is evident when we compared the microscopic structures of the conidiophores of *G. eximia* (Figure 6D) with the conidiophores of *T. gibellulae* (*G. pulchra* showing asexual and sexual states; Figure 6C) and *G. alata* (Figure 6E).

The host spiders that were morphologically identified comprised 15 families parasitised by 21 different fungal species (Figure 7). Some families of spiders are parasitised by more than one fungal species, such as Thomisidae, Anyphaenidae, Salticidae, Linyphiidae, Theridiidae, and Corinnidae [5,15,18,20,69]. As the fungus almost completely covers the host body, most studies were not able to identify the hosts at species rank. The 15 spider families belong to 10 distinct guilds, which comprise different ecological functions: (i) aerial space web builders (Theridiidae and Agelenidae), (ii) ground and aerial ambushers (Thomisidae), (iii) ground hunters (Zodariidae and Lycosidae), (iv) aerial hunters (Oxyopidae), (v) aerial ambushers (Sparassidae), (vi) ground weavers (Deinopidae), (vii) ground orb weavers (Linyphiidae), (viii) sedentary sheet weavers (Pholcidae), (ix) aerial runners (Anyphaenidae, Corinnidae, and Salticidae), and (x) aerial orb weavers (Tetragnathidae and Uloboridae) [70]. Araneidae was detected as a *Gibellula* host in reviews [5,31], but after checking the primary research, we did not find any precise record.

## 4. Discussion

### 4.1. Currently Accepted Species and Morphological Traits

**Figure 2:**
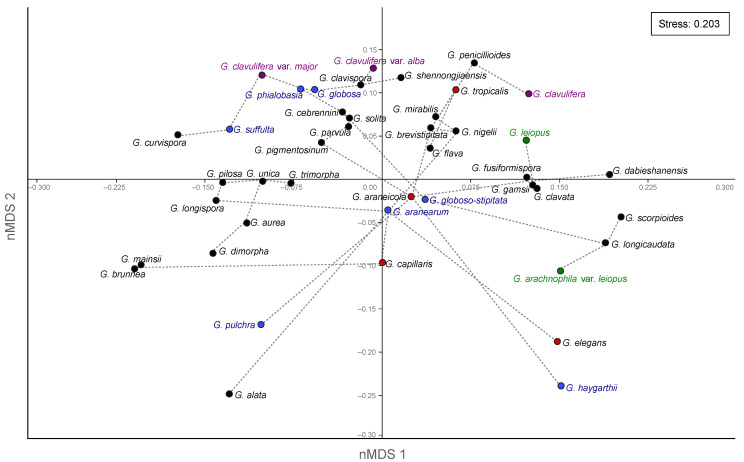
Non-Metric Dimensional Scaling (nMDS) performed using the morphological data of species descriptions for the genus *Gibellula*. Species that remain with the same name of original descriptions (accepted) are in black while species that were considered invalid are in red. The forms described for *G. clavulifera* are highlighted in purple, showing their intraspecific variation. The descriptions that were synonymised as *G. leiopus* are shown in green and those synonymised as *G. pulchra* are shown in blue, indicating a high variation among morphological characters. Grey lines show the Minimum Spanning Tree.

**Figure 3:**
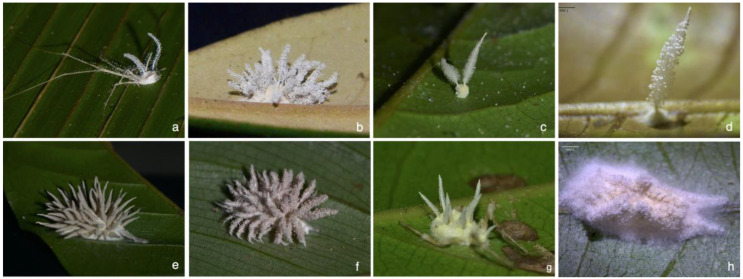
Representative species of *Gibellula* from the Brazilian Atlantic rainforests included in the molecular phylogeny of the genus. (**a**,**b**) *Gibellula pulchra* on spider (**a**) Pholcidae, (**b**) host not identified; (**c**,**d**) *Gibellula mirabilis*, hosts not identified; (**e**–**g**) *Gibellula leiopus,* hosts not identified; (**h**) *Gibellula mainsii*, host not identified.

**Figure 4:**
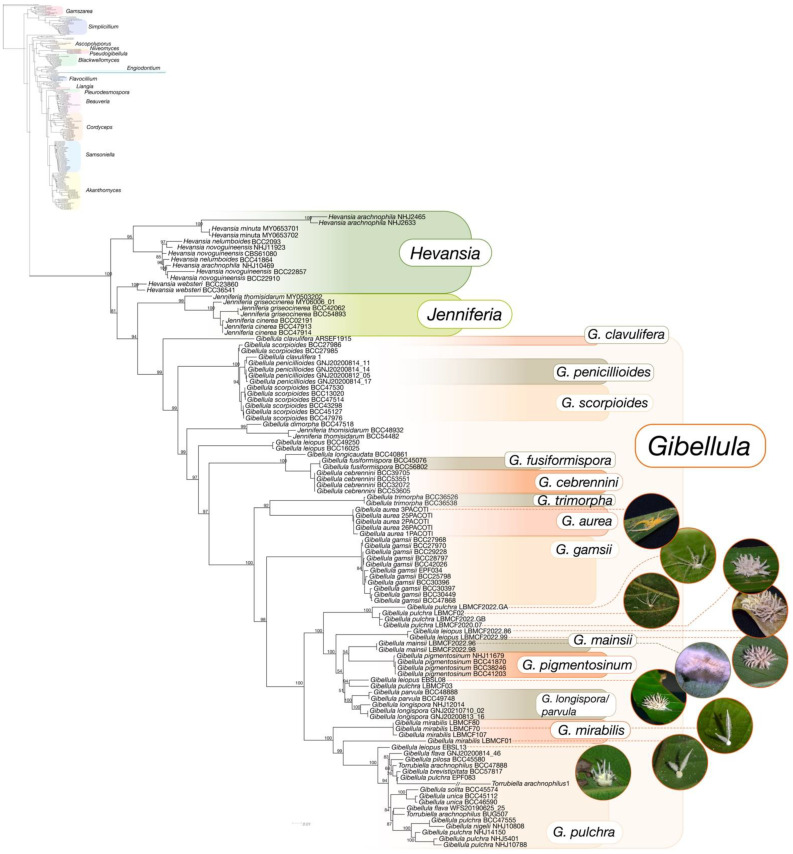
Phylogenetic tree using the Maximum Likelihood approach shows the specimens of *Gibellula* available at GenBank-NCBI databases, 14 fungal specimens collected at 4 Atlantic Rainforest sites in Brazil, and 21 genera of Cordycipitaceae, totalling 307 taxa. The analysis was based on concatenated sequences of SSU, ITS, LSU, RPB1, RPB2, and TEF markers. Numerical values on branches indicate the percentages of bootstraps. Bars show nucleotide substitutions per site.

**Figure 5:**
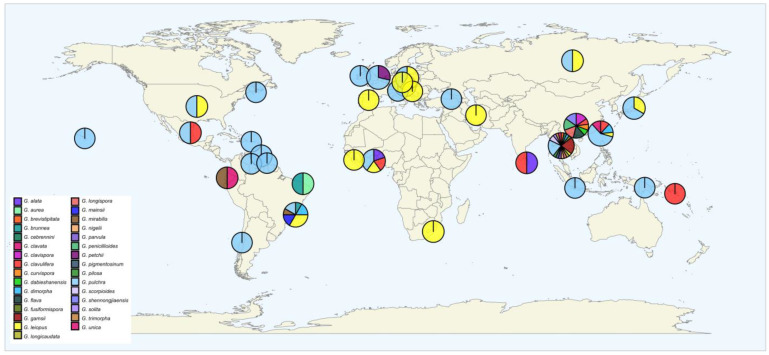
Global distribution of the fungus *Gibellula* infecting spiders. Species shown at the bottom left are the ones considered as accepted species in this review. Location was extracted from the 57 papers included in our databases.

**Figure 6:**
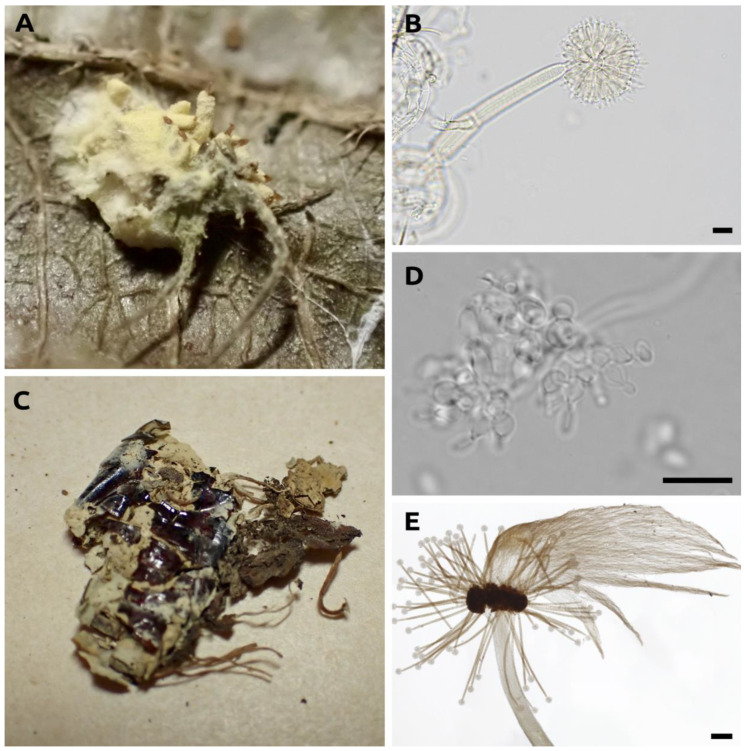
Type specimens of *Gibellula alata*, *Gibellula eximia*, and *T. gibellulae* (synonymized to *G. pulchra*). (**A**) Specimen of *T. gibellulae* (= *G. pulchra*); (**B**) conidiophore of *T. gibellulae* aspergillate, the most common shape for the genus; (**C**) Specimen of *G. eximia*; (**D**) conidiophore of *G. eximia* with different shape of conidiogenic cells; and (**E**) wing-like synnema of *G. alata* bearing aspergillate conidiophores. Microscopic structures were photographed in light microscopy. Scales: (**B**,**D**): 10 µm; (**E**) 200 µm.

**Figure 7:**
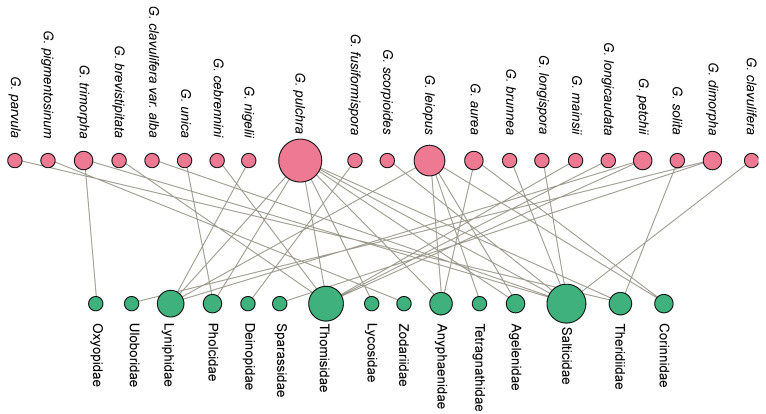
Bipartite network connecting the species of *Gibellula* (pink) and families of spiders (green) that were reported as hosts. Nodes (species/family) with higher numbers of connections are proportionally larger than the ones with fewer parasite–host associations.

The taxonomic rearrangements on *Gibellula* sp. were based on a large variation in morphological structures, as well as a wide geographic distribution of these species. These might indicate morphological variations among the populations, which led to several descriptions over time. For instance, *G. clavulifera* is proposed as four different variations (i.e., *G. clavulifera*, *G. clavulifera* var. *clavulifera*, *G. clavulifera* var. *alba*, *G. clavulifera* var. *major*) and there is morphological variance in the different varieties (Figure 2). However, morphological traits displayed in the nMDS cannot be considered as singular evidence to split the species as independent lineages due to the lack of a consistent standardised description that allows the comparison of all taxa, which resulted in a high value of stress on the analyses. When we investigated the molecular traits described for *G. clavulifera*, we detected that the high number of nucleotide substitutions per site corroborates their morphological differences and split in three distinct nodes from each other (Figure 4). Thus, evidence generated so far indicates that the varieties might be considered as different species; however, further taxonomic studies including the study of reference specimens and reference sequences are needed. The morphological traits extracted from our review (n = 19; Table S3) were the most frequent in the literature, appearing in at least 70% of the reviewed descriptions. The inclusion of these characters (including the specific terms referring to them) and their states (including absence) in future morphological descriptions would represent good practice for achieving a standard for describing *Gibellula* species.

Other taxa, such as *G. leiopus,* were reviewed to accommodate four species [19,24,31,62], and *G. pulchra* had 11 names of different species that were later associated with only 1 [20,24,27,31] (Figure 1). With these taxonomic proposals, a wide range of morphological traits are included in those species and assumed synonyms (e.g., *G. globosa* and *G. suffulta*) are eventually more distant than accepted distinct species in nMDS analysis (e.g., *G. unica* and *G. clavata*; Figure 2). Based on morphological and phylogenetic traits, we suggest that 31 of the published species of the genus *Gibellula* be accepted.

### 4.2. Multigene Phylogeny

The 14 specimens from formerly described species we present here are a major contribution to the hidden diversity of *Gibellula* in Atlantic Forests since we added information for the most spatially distributed species and filled an important gap to improve our phylogenetic inferences regarding the evolution of the genus. Nevertheless, the lack of molecular data for several species (22 from 54 records) still indicates the extent of the under-representation of the genus in public databases, with the RPB2 coding region being the most under-represented. For both ML and BI analyses, trees displayed the same topologies for most clades, but bootstrap percentages were low (less than 50%) for several clades and under the threshold considered as good support of the nodes [71]. Unfortunately, the lack of molecular data for both *G. petchii* and *G. aranearum* limited the evaluation of these taxa. Although the genus *Granulomanus* was suppressed [27], there is no molecular data specifically for *Granulomanus* to support this change. Therefore, we considered that the name *G. petchii* should be retained until the molecular description is performed. Surprisingly, *G. formosana* 1 is positioned outside the clade of *Gibellula* and among *Cordyceps* spp., which is a hint of misidentification of this specimen. Moreover, it is worth noting that species published before the year 2000 do not usually show molecular data [18,19,20]. This gap in molecular data for the genus hinders the precision of molecular identification as well as the inference of phylogenetic relationships.

### 4.3. Global Distribution

The geographic distribution of the genus *Gibellula* comprised five continents, in which species were widely reported in tropical and temperate regions. As most of the interactions were mainly reported in taxonomic-based descriptions, the ecological aspects are limited to fungal distribution and fungal diversity at specific locations [5,18,24]. One of the methods to mitigate this lack of ecological pattern is to estimate the potential distribution range of the genus and its ecological niche characterization [72,73,74]. For this, developing a model associating the current knowledge regarding the *Gibellula* sp. occurrence to an investigation of the distribution of the known hosts would help to predict the potential distribution and aspects of the biogeography of these spider–fungus interactions. As might be expected, the collected data so far might be influenced by sampling bias [75], which is usually strongly correlated to the presence of mycologists in a given area. Interestingly, there is no record of *Gibellula* in Australia, a country with a strong mycological community composed of both specialists and amateurs. Considering that *Gibellula* spp. are important pathogens for spider populations, this spatial distribution, identification of species, and evolutionary biological investigation can also be relevant for the molecular epidemiology monitoring of the genus.

### 4.4. Host–Parasite Interaction

The detection of 15 different host families of spiders parasitised by *Gibellula* sp. indicated that *Gibellula* species parasitise spiders from distinct phylogenetic origins and several ecological functions. For instance, *G. pulchra* parasitised spiders from lower to more recent clades [76], such as Pholcidae [5] and Salticidae [60], respectively. Furthermore, *G. pulchra* parasitises phylogenetically closer families, such as Lycosidae [77] and Thomisidae [78], even though the spiders belong to distinct guilds, considering that Lycosidae are generally ground hunters while Thomisidae are ground ambushers [70]. These spiders are not exclusively parasitised by *G. pulchra*. In addition, other two species of fungus are associated with Anyphaenidae and Corinnidae [5,15]. This association with more than a spider family demonstrates a lack of host specificity by the fungal parasite, which might suggest host-jumping events in spider–*Gibellula* interactions. Host-shifting is a strategy formerly identified in other entomopathogenic fungi that present similar transmission strategies within Clavicipitaceae [79], Cordycipitaceae [80], and Ophiocordycipitaceae [8], with different levels of host specificity [9,74]. Regarding *Gibellula* sp., even though the genus is reported as a specific parasite of spiders [16], it is still necessary to investigate the level of host specificity and whether these fungi have interkingdom host-jumping (parasitising animals and colonising plants as endophytes, for example [81]), which can influence their strategies to conquer the host body and the success of these interactions.

Despite this diversity of host spiders parasitised by *Gibellula*, the place of death of all these interactions is reported as the spiders attached to vegetation, especially underneath leaves. This reveals consistent behaviour regardless of the parasite or host species, but the suitability of the place for parasite development must be investigated. This extended phenotype has been experimentally demonstrated for arthropod–fungal interactions involving different phyla. In the ‘zombie-ants’ complex, ants die fixed by their legs or mandibles, in which fungi (Ascomycota) are unable to develop if the host ant is moved to the forest floor [1] or within its colony [82]. Within Entomophthoromycota, species of *Pandora* sp. [83] and *Entomophthora* sp. [84,85] induce changes in body posture before host death, and *Massospora* sp. interferes in host flight dynamics to disperse fungal spores [13]. Moreover, some genera also produce fungal structures to enhance host stability on the substrate, such as *Erynia* sp. in aphids [3] and *Entomophaga* sp. in locusts [86]. It is noteworthy that these examples strongly suggest that the place of death is a determinant of parasite ecological success. Therefore, based on the locations where spiders parasitised by *Gibellula* sp. are found on vegetation, this extended phenotype seems to be an important trait that should be investigated and might be a hint about the evolution of *Gibellula* sp. as a parasite of several spiders.

## Data Availability

All data generated or analysed during this study are included in this published article (and its supplementary information files). Alignments and phylogenetic trees generated for this study can be found in the Github repository https://github.com/LBMCF/gibellula_review.

## Supplementary Materials

The following supporting information can be downloaded at: https://www.mdpi.com/article/10.3390/jof9040457/s1, Figure S1: Global view of the records through database searching using the PRISMA diagram; Table S1: Primers used for amplification of each genomic region to identify the specimens of *Gibellula* sp.; Table S2: PCR conditions for amplification of each genomic region to identify the specimens of *Gibellula*; Table S3: Morphological characters from the original descriptions of *Gibellula* species; Table S4: Specimens from *Gibellula* sequenced in this study (LBMCF) and sequences of *Gibellula* species and Cordycipiceae from NCBI-GenBank databases used in phylogenetic analyses and their associated voucher numbers; Table S5: Nucleotide substitution models and partitions selected based on Bayesian Information Criterion; Figure S2: Phylogenetic analysis of the genus *Gibellula* using Maximum Likelihood with 1000 bootstraps with the dataset of the Internal Transcribed Spacer (ITS) region; Figure S3: Phylogenetic analysis of the genus *Gibellula* using Bayesian Inference with 30 million generations with the concatenated dataset of the six genomic regions.
