# Supplementary material for: Disentangling the Taxonomy, Systematics, and Life History of the Spider-Parasitic Fungus Gibellula (Cordycipitaceae, Hypocreales)
Source: J Fungi (Basel). 2023 Apr 8;9(4):457. doi: 10.3390/jof9040457 (PMC10146611; doi:10.3390/jof9040457)
Supplement: Supplementary file 1 [file jof-09-00457-s001.zip › TMP_Supplementary_materialV2.pdf]

Disentangling the taxonomy, systematics and life history of the spider-parasitic fungus *Gibellula* (Cordycipitaceae, Hypocreales)

Supplementary Materials

PRISMA 2020 flow diagram for new systematic reviews which included searches of databases, registers and other sources

Identification of studies via databases and registers

Identification

Records identified from*:
Databases (n = 4381)
Scopus (n = 774)
Jstor (n = 2905)
Web of Science (n = 702)

Records removed *before screening*:
Main subject not related to the topic of the review (n = 4171)

Screening

Records screened
(n = 210)

Records excluded**
Duplicate records removed
(n = 142)

Reports sought for retrieval
(n = 68)

Reports not retrieved:
Only abstract available (n = 1)

Reports assessed for eligibility
(n = 67)

Reports excluded:
Different taxa (n = 26)
Not primary research (n = 6)
Without fungal description (n = 9)

Included

Studies included in review
(n = 26)
Reports of included studies
(n = 30)

Identification of studies via other methods

Records identified from:
Citation searching (n = 31)

Reports sought for retrieval
(n = 31)

Reports not retrieved
(n = 1)

Reports assessed for eligibility
(n = 30)

Reports excluded:
(n = 0)

Figure S1. Global view of the records through database searching. The PRISMA diagram shows the main results of the searches for papers that presented interactions between entomopathogenic fungi and spiders, and the fungal morphological description. We conducted the literature searches from July to November 2022 at the databases *Web of Science – Core collection*, *Scopus* (Elsevier), and *JSTOR*, according to the suggested flow-chart for the literature selection process by PRISMA protocol [1].

Table S1. Primers used for amplification of each genomic region to identify the specimens of *Gibellula* sp.

Genomic region	Estimated size (bp)	Primers (sequence 5' → 3')	References
SSU	900-1000	NS1 (GTAGTCATATGCTTGTCTC)	[2]
		NS4 (CTTCCGTCAATTCCTTTAAG)	
ITS	600-700	ITS5 (GGAAGTAAAAGTCGTAACAAGG)	[2]
		ITS4 (TCCTCCGCTTATTGATATGC)	
LSU	800-900	LR7 (TACTACCACCAAGATCT)/LR5 (ATCCTGAGGGAAACTTC)	[3]
		LR0R (ACCCGCTGAACTTAAGC)	[4]
TEF	700-950	2218R (ATGACACCRACRGCRACRGTYTG)	[5]
		983F (GCYCCYGGHCAYCGTGAYTTYAT)	
RPB1	600-800	RPB1-Ac (GARTGYCCDGGDCAYTTYGG)	[6]
		RPB1-Cr (CCNGCDATNTCRTTRTCCATRTA)	
RPB2	900-1000	fRPB2-5F (GAYGAYMGWGATCAYTTYGG)	[7]
		fRPB2-7cR (CCCATRGCTTGTYYRCCCAT)	

Table S2. PCR conditions for amplification of each genomic region to identify the specimens of *Gibellula*

Genomic regions	Denaturation	Annealing 1	Annealing 2	Final extension	References
**SSU and LSU**	95 °C for 2 min	4 × (95 °C for 30 s, 55 °C for 30 s, 72 °C for 1 min)	35 × (95 °C for 30 s, 50.5 °C for 30 s, 72 °C for 1 min)	72 °C for 5 min	[8]

**ITS**	95 °C for 2 min	35 × (95 °C for 1min, 58 °C for 30 s, 72 °C for 1 min)	–	72 °C for 5 min	[2]
**TEF and RPB1**	95 °C for 2 min	10 × (95 °C for 30 s, 64 °C for 30 s, 72 °C for 1 min)	35 × (95 °C for 30 s, 54 °C for 30 s, 72 °C for 1 min)	72 °C for 5 min	[8]
**RPB2**	95 °C for 10 min	40 × (95 °C for 30s, 51.9 °C for 30s, 72 °C for 1min)	–	72 °C for 10 min	[7] adapted

Table S3. Morphological comparison of structures from specimens without molecular data available collected at the Brazilian Atlantic Rainforest and the original descriptions of *Gibellula* sp. used for nMDS analyses (available at the Github repository https://github.com/LBMCF/gibellula_review/TableS3_morphologicaltraits).

Table S4. Specimens from *Gibellula* sequenced in this study (LBMCF in bold) and sequences of *Gibellula* and Cordycipiceae species from NCBI-GenBank databases used in phylogenetic analyses and their associated voucher numbers. * Type species; **Lectotype species.

**Species name on GenBank**	**Voucher numbers**	**GenBank Accession Numbers**					
		**RPB2**	**RBP1**	**SSU**	**ITS**	**TEF**	**LSU**
Gibellula mirabilis	**LBMCF2020.01**	n/a	n/a	OQ585782	OQ589479	OQ658385	OQ585972
G. pulchra	**LBMCF2020.02**	n/a	n/a	OQ585783	n/a	OQ658386	OQ585973
G. pulchra	**LBMCF2020.03**	n/a	n/a	OQ585784	OQ589480	OQ658387	OQ585974
G. pulchra	**LBMCF2020.07**	n/a	n/a	OQ585785	n/a	OQ658388	OQ585975
G. mirabilis	**LBMCF2021.70**	n/a	n/a	OQ585786	OQ589481	OQ658389	OQ585976
G. mirabilis	**LBMCF2021.80**	n/a	n/a	OQ585787	OQ589482	OQ658390	OQ585977
G. leiopus	**LBMCF2022.86**	n/a	n/a	OQ585788	OQ589483	OQ658391	n/a
G. mainsii	**LBMCF2022.96**	n/a	n/a	OQ585789	OQ589484	OQ658392	n/a
G. leiopus	**LBMCF2022.98**	n/a	n/a	OQ585790	OQ589485	OQ658393	n/a
G. leiopus	**LBMCF2022.99**	n/a	n/a	OQ585791	OQ589486	OQ658394	OQ585978
G. mirabilis	**LBMCF2022.107**	n/a	n/a	OQ585792	n/a	OQ658395	OQ585979
G. leiopus	**EBSL08 (LBMCF0011)**	OL117024	n/a	OK329882	OQ589488	OK392621	OK329878
G. leiopus	**EBSL13 (LBMCF0001)**	n/a	n/a	OK329879	OK329883	OK392625	OK329874

G. pulchra	**LBMCF2022.GA**	n/a	n/a	OQ585780	n/a	OQ658383	OQ585970
G. pulchra	**LBMCF2022.GB**	n/a	n/a	OQ585781	OQ589487	OQ658384	OQ585971
Gibellula aurea	1PACOTI (LBMCF0003)	OL117022	n/a	OK329880	n/a	OK392618	OQ585967
G. aurea	2PACOTI (LBMCF0004)	OL117023	n/a	OK329881	n/a	OK392619	OQ585968
G. aurea	3PACOTI (LBMCF0005)	n/a	n/a	n/a	n/a	OQ658382	OQ585969
G. aurea	25PACOTI (LBMCF0006)	OK315662	n/a	n/a	n/a	OK392624	OK329875
G. aurea	26PACOTI (LBMCF0007)	OK315663	n/a	n/a	OK329885	OK392622	OK329876
G. brevistipitata	BCC57817	n/a	OK040715	n/a	OK040729	OK040697	OK040706
G. cebrennini	BCC32072	n/a	n/a	n/a	MT477067	MT503326	n/a
G. cebrennini	BCC53551	n/a	n/a	n/a	MT477068	MT503327	n/a
G. cebrennini	BCC53605	MT503336	MT503321	n/a	MT477069	MT503328	MT477062
G. cebrennini	BCC39705	MH521859	MH521822	n/a	MH532874	MH521895	MH394673
G. clavulifera	GZUIFR-HN0801	n/a	n/a	n/a	KJ857269	n/a	n/a
G. clavulifera	1	n/a	n/a	n/a	KP685596	n/a	n/a
G. clavulifera var. alba	ARSEF1915	DQ522467	DQ522408	DQ522562	JN049837	DQ522360	DQ518777
G. dimorpha	BCC47518	MH521863	MH521819	n/a	MH532884	MH521892	MH394679
G. flava	GNJ20200814-46	n/a	MW980146	MW969660	n/a	MW961413	MW969673
G. flava	WFS20190625-25	n/a	MW384883	MW036749	n/a	MW091325	MW084343
G. formosana	1	n/a	n/a	n/a	AB100360	n/a	n/a
G. fusiformispora	BCC56802	MT503337	MT503322	n/a	MT477070	MT503329	MT477063
G. fusiformispora	BCC45076	MH521860	MH521823	n/a	MH532882	n/a	n/a
G. gamsii	BCC25798	n/a	MH152550	n/a	MH152532	MH152563	MH152542
G. gamsii	BCC27968	n/a	MH152547	n/a	MH152529	MH152560	MH152539
G. gamsii	BCC27970	n/a	MH152548	n/a	MH152530	MH152561	MH152540
G. gamsii	BCC28797	MH152557	MH152549	n/a	MH152531	MH152562	MH152541
G. gamsii	BCC29228	MH152558	MH152551	n/a	MH152533	MH152564	MH152543
G. gamsii	BCC30396	n/a	MH152553	n/a	MH152535	MH152566	n/a

G. gamsii	BCC30397	n/a	MH152554	n/a	MH152536	MH152567	n/a
G. gamsii	BCC30449	MH152559	MH152552	n/a	MH152534	MH152565	MH152544
G. gamsii	BCC42026	n/a	MH152555	n/a	MH152537	MH152568	MH152545
G. gamsii	BCC47868	MH152556	MH152546	n/a	n/a	n/a	MH152538
G. gamsii	EPF034	n/a	n/a	n/a	JX192720	JX192817	JX192753
G. leiopus	BCC 16025	n/a	MF416649	MF416602	n/a	MF416492	MF416548
G. leiopus	BCC49250	OK070784	OK070783	n/a	OK070780	OK070782	OK070781
G. longicaudata	BCC40861	OK040724	OK040716	n/a	OK040730	OK040698	OK040707
G. longispora	GNJ20210710-02	OL981635	n/a	OL854201	n/a	OL981628	OL854212
G. longispora	GNJ20200813-16	n/a	MW980145	n/a	n/a	MW961414	n/a
G. longispora	NHJ 12014	EU369075	EU369055	EU369098	n/a	EU369017	n/a
G. nigelii	NHJ 10808	EU369076	EU369056	EU369099	n/a	EU369018	EU369035
G. parvula	BCC48888	OK040725	OK040717	n/a	NR_182399	OK040699	OK040708
G. parvula	BCC49748	OK040726	OK040718	n/a	OK040732	OK040700	OK040709
G. penicillioides	GNJ20200812-05	n/a	n/a	n/a	MW969672	MW961418	MW969664
G. penicillioides	GNJ20200814-11	n/a	MZ215998	MW969650	MW969669	MW961415	MW969661
G. penicillioides	GNJ20200814-14	n/a	MZ215999	MW969651	MW969670	MW961416	MW969662
G. penicillioides	GNJ20200814-17	n/a	n/a	MW969652	MW969671	MW961417	MW969663
G. pigmentosinum	BCC41203	n/a	MT503323	n/a	MT477071	MT503330	n/a
G. pigmentosinum	BCC41870	n/a	MT503324	n/a	MT477072	MT503331	MT477064
G. pigmentosinum	BCC38246	MH521855	MH521800	n/a	MH532872	MH521893	MH394672
G. pigmentosinum	NHJ 11679	n/a	EU369054	n/a	n/a	EU369016	n/a
G. pilosa	BCC45580	n/a	OK040719	n/a	OK040733	OK040701	OK040710
G. pulchra	NHJ 10788	EU369078	EU369058	EU369101	n/a	EU369019	EU369036
G. pulchra	NHJ 5401	EU369079	EU369059	EU369102	n/a	n/a	n/a
G. pulchra	BCC47555	n/a	MH521804	n/a	MH532885	MH521897	n/a
G. pulchra	NHJ14150	n/a	n/a	n/a	HM161739	HM161729	n/a
G. pulchra	EPF083	n/a	n/a	JX192782	JX192719	JX192813	JX192752
G. scorpioides	BCC45127	n/a	n/a	n/a	MT477075	MT503332	n/a
G. scorpioides	BCC47514	n/a	n/a	n/a	MT477076	MT503333	n/a
G. scorpioides	BCC47530	MT503338	n/a	n/a	MT477077	MT503334	MT477065
G. scorpioides	BCC47976	MT503339	MT503325	n/a	MT477078	MT503335	MT477066

G. scorpioides	BCC13020	n/a	MH521814	n/a	MT477073	MH521901	MH394686
G. scorpioides	BCC27985	MH521857	MH521815	n/a	n/a	MH521899	MH394662
G. scorpioides	BCC27986	OK040727	OK040720	n/a	OK040735	OK040702	OK040711
G. solita	BCC45574	n/a	OK040721	n/a	OK040736	OK040703	OK040712
G. trimorpha	BCC36538	MH521861	MH521817	n/a	MH532867	MH521890	MH394668
G. trimorpha	BCC36526	OK040728	OK040722	n/a	OK040737	OK040704	n/a
G. unica	BCC46590	MH521866	MH521803	n/a	MH532883	n/a	MH394678
G. unica	BCC45112	n/a	OK040723	n/a	OK040738	OK040705	OK040713
Torrubiella arachnophilus (G. pulchra)	BCC47888	MH521864	MH521802	n/a	n/a	n/a	n/a
T. arachnophilus	1	n/a	n/a	AF327399	KP685595	n/a	AF327391
Torrubiella arachnophilus	BUG507	MH879619	MH885445	MH879644	n/a	n/a	MH879596
Akanthomyces aculeatus	TS772	n/a	n/a	KC519368	n/a	KC519366	KC519370
A. araneicola	GY 29011	MK955947	MK955944	n/a	MK942431	MK955950	n/a
A. araneicola	GY 29012	MK955948	MK955945	n/a	MK942434	MK955951	n/a
A. attenuatus	CBS 170.76	OP762615	OP762611	n/a	MH872739	OP762607	OP752153
A. attenuatus	CBS402.78	EF468935	EF468888	AF339614	AJ292434	EF468782	AF339565
A. attenuatus	KACC42493	KM283846	KM283826	KM283756	n/a	KM283804	KM283780
A. attenuatus	KACC43049	KM283847	KM283827	KM283757	n/a	KM283805	KM283781
A. coccidioperitheciatus	NHJ 6709	EU369086	EU369067	EU369110	JN049865	EU369025	EU369042
A. kanyawimiae	BCC 34340	MH521875	MH521831	n/a	MH532862	MH521909	MH394666
A. kanyawimiae	TBRC 7242	MF140808	MF140784	n/a	MF140751	MF140838	MF140718
A. lecanii	CBS101247	KM283859	KM283837	KM283770	JN049836	DQ522359	KM283794
A. lecanii	CBS102067	KM283860	KM283838	KM283771	n/a	KM283818	KM283795
A. neocoleopterorum	GY11241	MN097812	MN097816	n/a	MN093295	MN097813	n/a
A. neocoleopterorum	GY11242	MN097814	MN097817	n/a	MN093297	MN097815	n/a
A. noctuidarum	BBH16595	MT478005	MT477995	n/a	MT356073	MT477979	MT356085
A. noctuidarum	BCC28571	MT478006	MT478009	n/a	MT356075	MT477981	MT356087
A. pyralidarum	BCC29197	MT477991	MT478003	n/a	MT356083	MT508840	MT356094
A. pyralidarum	BCC40869	MT477990	MT478002	n/a	MT356082	MT477984	MT356093

A. sulphureus	TBRC 7247	MF140811	MF140785	n/a	MF140756	MF140841	MF140720
A. sulphureus	TBRC 7248	MF140812	MF140787	n/a	MF140758	MF140843	MF140722
A. thailandicus	TBRC 7245	MF140809	n/a	n/a	MF140754	MF140839	n/a
A. thailandicus	TBRC 7246	MF140810	n/a	n/a	MF140755	MF140840	MF140719
A. tortricidarum	BCC41868	MT478008	MT477998	n/a	MT356077	MT477985	MT356089
A. tortricidarum	BCC72638	MT477992	MT477997	n/a	MT356076	MT478004	MT356088
A. tuberculatus	BCC12869	n/a	n/a	GQ249961	GQ250007	GQ250036	GQ249986
A. tuberculatus	BCC16819	n/a	n/a	GQ249962	GQ250012	GQ250037	GQ249987
A. waltergamsii	TBRC 7251	MF140833	MF140781	n/a	MF140747	MF140833	MF140713
A. waltergamsii	TBRC 7252	MF140806	MF140782	n/a	NR_164417	MF140834	MF140714
*A. aculeatus**	BCC 17075	n/a	n/a	GQ249958	GQ250011	GQ250033	GQ249983
Ascopolyporus albus	BCC48976	OL322066	OL322057	n/a	OL331503	OL322036	OL322049
Asc. galloides	BCC25446	OL322060	OL322053	n/a	OL331510	OL322029	OL322042
Asc. galloides	BCC47981	OL322061	OL322054	n/a	OL331511	OL322030	OL322043
Asc. griseoperitheciatus	BCC22358	OL322067	n/a	n/a	OL331507	OL322037	OL322050
Asc. griseoperitheciatus	BCC25788	OL322068	OL322058	n/a	OL331508	OL322038	OL322051
Asc. purpuratus	BCC88388	OL322064	n/a	n/a	OL331505	OL322033	OL322046
Asc. purpuratus	BCC88430	OL322063	OL322059	n/a	OL331506	OL322032	OL322045
Asc. albus	BCC48975	OL322065	OL322056	n/a	OL331502	OL322035	OL322048
Beauveria acridophila	MCA 1181	n/a	MF416628	MF416574	n/a	n/a	MF416522
B. amorpha	ARSEF 4149	HQ880948	HQ880876	n/a	HQ880804	HQ881006	n/a
B. asiatica	BUB824	MG642878	MG642863	MG642893	MG642836	MG642908	MG642850
B. australis	ARSEF 4622	HQ880934	HQ880862	n/a	HQ880790	HQ880996	n/a
B. australis	ARSEF 4580	HQ880932	HQ880860	n/a	HQ880788	HQ880994	n/a
*B. bassiana***	ARSEF 1564	HQ880905	HQ880833	n/a	GU734762	HQ880974	n/a
B. brongniartii	ARSEF 6215	HQ880925	HQ880853	n/a	HQ880781	HQ880990	n/a
B. brongniartii	ARSEF 7058	HQ880917	HQ880845	n/a	HQ880773	HQ880983	n/a
B. brongniartii	ARSEF 7268	HQ880916	HQ880844	n/a	HQ880772	HQ880982	n/a
B. brongniartii	BCC 16585	JF415991	JN049885	JF415951	JN049867	JF416009	JF415967
B. caledonica	ARSEF 2567	HQ880961	EF469086	AF339570	HQ880817	EF469057	AF339520
B. caledonica	BUB421	MG642873	MG642858	MG642888	MG642831	MG642903	MG642845
B. locustiphila	TS 881	JX003845	JX003847	JQ895525	JQ958606	JQ958619	JQ895535

B. malawiensis	BCC 20195	MN401602	MN401546	n/a	MN401622	MN401448	n/a
B. malawiensis	BUB444	MG642875	MG642860	MG642890	MG642833	MG642905	MG642847
B. pseudobassiana	BUB506	MG642876	MG642861	MG642891	MG642834	MG642906	MG642848
B. pseudobassiana	YNHHCS1	MH458410	MH458402	MH458406	n/a	MH458404	MH458408
B. scarabaeidicola	ARSEF 5689	DQ522431	DQ522380	AF339574	AY245639	DQ522335	AF339524
B. sinensis	BUB51	MG642864	MG642851	MG642879	MG642824	MG642894	MG642837
B. amorpha	ARSEF 7542	HQ880949	HQ880877	n/a	HQ880805	HQ881007	n/a
Blackwellomyces aurantiacus	BCC 85061	MT017820	MK411601	n/a	MT000693	MK411599	MT003029
Bla. calendulinus	BCC68500	MT017821	MT017802	n/a	MT000694	MT017842	MT003030
Bla. calendulinus	BCC68502	MT017822	MT017803	n/a	MT000695	MT017843	MT003031
Bla. cardinalis	OSC 93610	EF469106	EF469088	AY184973	JN049843	EF469059	AY184963
*Bla. cardinalis**	OSC 93609	DQ522422	DQ522370	AY184974	n/a	DQ522325	AY184962
Bla. lateris	MFLU 18-0663	MK079354	MK084615	NG_067678	NR_166258	MK069471	NG_067857
Bla. minutus	BCC 88269	MT017823	MT017804	n/a	MT000696	MT017844	MT003032
Bla. pseudomilitaris	NBRC 101409	n/a	JN992482	JN941748	JN943305	n/a	JN941393
Bla. pseudomilitaris	NBRC 101410	n/a	JN992481	JN941747	JN943307	n/a	JN941394
Bla. roseostromatus	BCC 91358	MT017824	MT017805	n/a	MT000697	MT017845	MT003033
Bla. roseostromatus	BCC 91359	MT017825	MT017806	n/a	MT000698	MT017846	MT003034
Bla. aurantiacus	BCC 85060	MT017819	MK411600	n/a	MT000692	MK411598	MT003028
Cordyceps araneae	BCC85066	MT017829	MT017811	n/a	MT000703	MT017851	MT003038
C. cateniobliqua	YFCC 3367	MN576935	MN576881	MN576765	n/a	MN576991	MN576821
C. cateniobliqua	YFCC 5935	MN576936	MN576882	MN576766	n/a	MN576992	MN576822
C. fumosorosea	CBS 107.10	MG665237	n/a	n/a	AY624184	n/a	MG665227
C. fumosorosea	CBS 375.70	MG665238	n/a	n/a	MH859721	n/a	MG665229
C. javanica	CBS 134.22	MF416455	MF416661	MF416610	MH854719	MF416504	MG665231
C. javanica	TBRC 7260	MF140803	MF140779	n/a	MF140744	MF140830	MF140710
C. lepidopterorum	TBRC 7263	MF140792	MF140768	n/a	MF140765	MF140819	NG_067804
C. lepidopterorum	TBRC 7264	MF140793	MF140769	n/a	MF140766	MF140820	MF140700
C. longiphialis	YFCC 8402	OL473536	OL739571	NG_148882	n/a	OL473525	OL468577
C. longiphialis	YFCC 8403	OL473537	OL739572	OL468558	n/a	OL473526	OL468578
C. militaris	OSC 93623	n/a	DQ522377	AY184977	JN049825	DQ522332	AY184966

C. nidus	TS903C	n/a	KY360296	KY360300	n/a	n/a	KY360293
C. pruinosa	ARSEF 5413	DQ522451	Q522397	AY84979	JN049826	DQ522351	AY84968
C. simaoensis	YFCC 8407	OL473541	OL739576	OL468562	n/a	OL473530	OL468582
C. simaoensis	YFCC 8408	OL473542	OL739577	OL468563	n/a	OL473531	OL468583
C. subtenuipes	YFCC 6051	MN576891	MN576835	MN576719	n/a	MN576945	MN576755
C. subtenuipes	YFCC 6084	MN576892	MN576836	MN576720	n/a	MN576946	MN576776
C. tenuipes	ARSEF 5135	JF416000	JN049896	MF416612	AY624196	KY973654	JF415980
C. tenuipes	BCC33299	MH521877	MH521838	n/a	MH532860	n/a	MH394664
C. tenuipes	BCC34337	MH521878	MH521839	n/a	MH532861	n/a	MH394665
C. araneae	BCC85065	MT017828	MT017810	n/a	MT000702	MT017850	MT003037
Engyodontium rectidentatum	CBS 641.74	n/a	n/a	n/a	LC092895	LC425540	LC092914
Eng. rectidentatum	CBS 547.82	n/a	n/a	n/a	LC092894	LC425544	LC092913
*Flavocillium bifurcatum**	YFCC 6101	MN576897	MN576841	MN576725	MN576833	MN576951	MN576781
Fla. subprimulinum	KUMCC 17-0144	n/a	n/a	MG585320	MG585318	MG585321	MG585319
Fla. subprimulinum	KUMCC 17-0148	n/a	n/a	MG585316	MG585314	MG585317	MG585315
Fla. subprimulinum	JCM 18525	n/a	n/a	NG_073501	NR_119418	LC557125	NG_067516
Fla. acerosum	CBS418.81	KM283852	KM283832	KM283762	EF641893	KM283810	KM283786
Gam. lunata	LC12546	MK335982	n/a	n/a	n/a	MK336030	n/a
Gamszarea microspora	CGMCC3.19313	n/a	n/a	NG_074898	NR_172832	n/a	NG_075269
Gam. microspora	LC12531	MK335984	n/a	n/a	n/a	MK336032	n/a
*Gam. wallacei**	CBS 101237	EF469119	EF469102	NG_062646	NR_111267	EF469073	NG_042398
Gam. humicola	LC12462	MK335980	n/a	n/a	n/a	MK336028	n/a
Hevansia arachnophila	NHJ2465	ON470207	ON470205	n/a	MH532899	MH521916	n/a
H. arachnophila	NHJ2633	MH521884	MH521843	n/a	MH532900	MH521917	n/a
H. cinerea	BCC02191	n/a	n/a	GQ249956	GQ250000	GQ250029	GQ249971
H. cinerea	BCC47914	MH521851	MH521821	n/a	n/a	MH521888	MH394652
H. cinerea	BCC47913	MH521850	MH521820	n/a	n/a	n/a	MH394651
H. koratensis	BCC01485	ON470208	ON470206	GQ249957	GQ250010	GQ250031	GQ249981
H. minuta	MY060537.01	MZ707833	MZ707826	n/a	n/a	MZ707811	n/a
H. minuta	MY060537.02	MZ707834	MZ707827	n/a	n/a	MZ707812	n/a

H. nelumboides	BCC2093	MF416437	n/a	MF416583	n/a	MF416473	MF416530
H. nelumboides	BCC 41864	n/a	n/a	JN201863	JN201871	JN201867	JN201873
H. novoguineensis	BCC22910	n/a	n/a	GQ249953	GQ250003	GQ250024	GQ249974
H. novoguineensis	NHJ11923	EU369072	EU369052	EU369095	n/a	EU369013	EU369032
H. novoguineensis	CBS 610.80	MH521844	n/a	n/a	MH532831	MH521885	MH394646
*H. novoguineensis**	BCC22857	n/a	n/a	GQ249952	GQ250002	GQ250023	GQ249973
H. websteri	BCC36541	MH521849	MH521811	n/a	MH532868	MH521889	MH394669
H. websteri	BCC23860	n/a	n/a	GQ249954	GQ250009	GQ250030	GQ249979
Hev. arachnophila	NHJ 10469	n/a	EU369047	EU369090	n/a	EU369008	EU369031
Jenniferia griseocinerea	BCC 54893	n/a	n/a	n/a	MZ684093	n/a	MZ684008
Jen. griseocinerea	MY06006.01	MZ707837	MZ707828	n/a	n/a	MZ707815	n/a
Jen. thomisidarum	BCC 48932	n/a	n/a	n/a	MZ684095	n/a	MZ684012
Jen. thomisidarum	BCC 54482	n/a	n/a	n/a	MZ684097	n/a	MZ684014
*Jen. thomisidarum**	MY05032.02	MZ707844	MZ707831	n/a	n/a	MZ707824	n/a
Jen. griseocinerea	BCC 42062	n/a	n/a	n/a	MZ684091	n/a	MZ684006
Lecanicillium aphanocladii	CBS797.84	KM283853	KM283833	KM283763	n/a	KM283811	KM283787
L. aphanocladii	IFM 64743	n/a	n/a	LC553289	LC553279	LC553294	LC553284
L. araneogenum	GZU1031Lea	KX845701	KX845699	KX845705	n/a	KX845697	KX845703
L. araneogenum	GZU1032Lea	KX845702	KX845700	KX845706	n/a	KX845698	KX845704
L. coprophilum	CGMCC 3.18986	n/a	n/a	NG_065751	NR_163303	n/a	NG_067818
L. coprophilum	TBS419	MH177623	MH177621	MH177626	MH177615	MH184586	MH177618
L. dimorphum	CBS345.37	KM283854	KM283834	KM283764	n/a	KM283812	KM283788
L. flavidum	CBS300.70D	KM283855	n/a	KM283765	MH859668	KM283813	KM283789
L. flavidum	CBS342.80	n/a	n/a	KM283766	EF641878	KM283814	KM283790
L. fungicola var. aleophilum	CBS357.80	KM283856	KM283835	KM283767	n/a	KM283815	KM283791
L. fusisporum	CBS164.70	KM283858	KM283836	KM283769	NR_111100	KM283817	AF339549
L. huhutii	GZUIFRhuhu	MT006063	MT006058	MN963916	MN944445	MT006068	n/a
L. longisporum	CBS102072	KM283861	KM283839	KM283772	n/a	KM283819	KM283796
L. longisporum	CBS126.27	KM283862	KM283840	KM283773	OP756342	KM283820	KM283797
L. magnisporum	CGMCC3.19304	n/a	n/a	NG_074899	NR_172833	n/a	NG_075270

L. muscarium	CBS 143.62	KM283863	KM283841	KM283774	n/a	KM283821	NG_058106
L. pissodis	BBC7	MT027509	MT027506	MT004819	MT004829	MT027503	MT004835
L. pissodis	CBS118231	KM283864	KM283842	KM283775	n/a	KM283822	KM283799
L. praecognitum	MGC39	MT267525	n/a	MT247062	MT247058	MT267523	MT247060
L. praecognitum	WA67215	n/a	n/a	NG_070677	NR_173935	n/a	NG_081473
L. psalliotae	CBS101270	EF469113	EF469095	AF339607	n/a	EF469066	AF339558
L. psalliotae	CBS532.81	EF469112	EF469096	AF339609	JN049846	EF469067	AF339560
L. tenuipes	CBS30985	DQ522439	KM283844	KM283778	JN036556	DQ522341	KM283802
L. uredinophilum	CEP 054	OP762612	OP762608	n/a	n/a	n/a	OP752150
L. antillanum	CBS350.85	DQ522450	DQ522396	AF339585	NR_111097	DQ522350	AF339536
Leptobacillium leptobactrum	CBS 774.69	n/a	n/a	n/a	MH859421	n/a	MH871192
*Lep. leptobactrum**	CBS 775.69	n/a	n/a	n/a	MH859422	n/a	MH871193
Lep. filiforme	URM 7918	n/a	n/a	n/a	NR_171744	n/a	NG_075252
Liangia sinensis	YFCC 3103	MN576898	MN576842	MN576726	MN576831	MN576952	MN576782
*Lia. sinensis**	YFCC 3104	MN576899	MN576843	MN576727	MN576832	MN576953	MN576783
*Microhilum oncoperae**	ARSEF 4358	EF468936	EF468891	AF339581	n/a	EF468785	AF339532
Neotorrubiella chinghridicola	BCC80733	MK632149	MK632176	MK632121	MK632039	n/a	MK632097
*Neo. chinghridicola**	BCC39684	MK632148	MK632181	MK632122	MK632038	MK632071	MK632096
Niveomyces coronatus	NY04434800	ON513400	ON513399	ON493547	n/a	ON513397	ON493606
Ophiocordyceps caloceroides	MCA 2249	n/a	MF416632	MF416578	n/a	MF416470	MF416525
O. gracilis	EFCC 8572	EF468912	EF468859	EF468956	JN049851	EF468751	EF468811
O. salganeicola	Mori01	MT759580	MT759578	MT741705	n/a	MT759575	MT741719
Parengyodontium album	CBS368.72	n/a	n/a	n/a	MH860502	LC382183	MH872217
Phytocordyceps ninchukispora	EGS 38.165	n/a	EF468900	EF468991	n/a	EF468795	EF468846
Phy.ninchukispora	EGS 38.166	n/a	EF468901	EF468992	n/a	EF468794	EF468847
*Pleurodesmospora coccorum**	CBS 460.73	n/a	n/a	n/a	MH860743	n/a	MH872455
Ple. lepidopterorum	DY10501	MW834316	MW834315	n/a	MW826577	MW834317	n/a

Ple. coccorum	CBS 459.73	n/a	n/a	n/a	MH860742	n/a	MH872454
Polystromomyces araneae	MY12684	MZ707845	MZ707832	n/a	n/a	MZ707825	n/a
Pseudogibellula formicarum	BCC 81493	n/a	MT533472	n/a	n/a	MT863566	MT512652
P. formicarum	BCC 84257	n/a	MT533473	n/a	n/a	MT533480	MT512653
P. formicarum	CBS 433.73	n/a	n/a	n/a	AY945231	n/a	n/a
P. formicarum	CBS 871.72	n/a	MT533474	n/a	n/a	MT863565	MH878295
Samsoniella alpina	YFCC 5818	MN576923	MN576869	MN576753	n/a	MN576979	MN576809
Sam. alpina	YFCC 5831	MN576924	MN576870	MN576754	n/a	MN576980	MN576810
Sam. antleroides	YFCC 6016	MN576917	MN576863	MN576747	n/a	MN576973	MN576803
Sam. antleroides	YFCC 6113	MN576918	MN576864	MN576748	n/a	MN576974	MN576804
Sam. aurantia	TBRC 7271	MF140818	MF140791	n/a	MF140764	MF140846	MF140728
Sam. aurantia	TBRC 7272	MF140817	n/a	n/a	MF140763	MF140845	MF140727
Sam. cardinalis	YFCC 5830	MN576902	MN576848	MN576732	n/a	MN576958	MN576788
Sam. cardinalis	YFCC 6144	MN576900	MN576846	NG_077413	n/a	MN576956	MN576786
Sam. coccinellidicola	YFCC 8772	ON568685	ON676502	ON563166	n/a	ON676514	ON621670
Sam. coccinellidicola	YFCC 8773	ON568686	ON676503	ON563167	n/a	ON676515	ON621671
Sam. cristata	YFCC 6023	MN576906	MN576852	MN576736	n/a	MN576962	MN576792
Sam. cristata	YFCC 7004	MN576907	MN576853	NG_077415	n/a	MN576963	MN576793
Sam. farinospora	YFCC 8774	ON568687	ON676504	ON563168	n/a	ON676516	ON621672
Sam. farinospora	YFCC 9051	ON568688	ON676505	ON563169	n/a	ON676517	ON621673
Sam. haniana	YFCC 8769	ON568689	ON676506	ON563170	n/a	ON676518	ON621674
Sam. haniana	YFCC 8770	ON568690	ON676507	ON563171	n/a	ON676519	ON621675
Sam. hepiali	YFCC 7024	MN576911	MN576857	MN576741	n/a	MN576967	MN576797
Sam. hepiali	YFCC 7215	MN576912	MN576858	MN576742	n/a	MN576968	MN576798
Sam. inthanonensis	TBRC 7270	MF140813	MF140788	n/a	MF140759	MF140847	MF140723
Sam. inthanonensis	TBRC 7915	MF140815	MF140790	n/a	MF140761	MF140849	MF140723
Sam. kunmingensis	YHH 16002	MN576916	MN576862	MN576746	n/a	MN576972	NG_079657
Sam. lanmaoa	YFCC 6193	MN576904	MN576850	MN576734	n/a	MN576960	MN576790
Sam. lanmaoa	YFCC 6148	MN576903	MN576849	MN576733	n/a	MN576959	MN576789
Sam. pseudotortricidae	YFCC 9052	ON568692	ON676509	ON563173	n/a	ON676521	ON621677

Sam. pseudotortricidae	YFCC 9053	ON568693	ON676510	ON563174	n/a	ON676522	ON621678
Sam. ramosa	YFCC 6020	MN576919	MN576865	MN576749	n/a	MN576975	MN576805
Sam. tortricidae	YFCC 6013	MN576921	MN576867	MN576751	n/a	MN576977	MN576807
Sam. tortricidae	YFCC 6131	MN576920	MN576866	NG_077418	n/a	MN576976	MN576806
Sam. yunnanensis	YFCC 1527	MN576926	MN576872	NG_077420	n/a	MN576982	MN576812
Sam. yunnanensis	YFCC 1824	MN576927	MN576873	MN576757	n/a	MN576983	MN576813
Sam. alboaurantia	BBC5	MT027508	n/a	MT004818	MT004827	MT027502	MT004834
Simplicillium aogashimaense	JCM 18167	n/a	n/a	NG_068378	NR_111026	LC496904	NG_068547
Sim. cylindrosporum	JCM 18169	n/a	n/a	NG_068379	NR_111023	LC496906	NG_069476
Sim. formicae	MFLUCC 18-1379	n/a	MK882623	NG_070121	NR_168789	MK926451	NG_068624
Sim. lamellicola	CBS 116.25	DQ522464	DQ522404	AF339601	NR_111098	DQ522356	NG_042381
Sim. lamellicola	JC-1	n/a	n/a	MT807908	MT807906	MT826785	MT807907
Sim. lanosoniveum	CG888	n/a	n/a	MT081951	MT081944	MT140365	MT081957
Sim. lanosoniveum	CG889	n/a	n/a	MT081952	MT081943	MT140366	MT081958
*Sim. lanosoniveum**	CBS 704.86	DQ522464	DQ522406	AF339602	n/a	DQ522358	AF339553
Sim. minatense	JCM 18176	n/a	n/a	NG_068380	NR_111025	LC496908	NG_069477
Sim. niveum	BCC83036	n/a	MW603489	n/a	MW621499	MW603488	MW620992
Sim. obclavatum	CBS 311.74	n/a	n/a	NG_062627	NR_111099	EF468798	NG_042535
Sim. subtropicum	JCM 18180	n/a	n/a	LC496895	NR_111024	LC496910	LC496880
Sim. sympodiophorum	JCM 18184	n/a	n/a	NG_068382	NR_111027	LC496912	NG_068548
Sim. yunnanense	YFCC 7133	n/a	MN576844	NG_077412	n/a	MN576954	MN576784
Sim. yunnanense	YFCC 7134	n/a	MN576845	MN576729	n/a	MN576955	MN576785
Sim. aogashimaense	JCM 18168	n/a	n/a	LC496890	AB604004	LC496905	LC496875

Table S5. Nucleotide substitution models and partitions selected based on Bayesian Information Criterion (BIC) in ModelFinder [9] and Partition Models [10].

Partition	Genomic region	Substitution Models	Sequences	Unique site patterns	Informative sites	Invariant sites	Constant sites
1	SSU	K2P+I+G4	164	476	222	720	720
2	LSU	SYM++I+G4	257	763	419	726	726
3	TEF- *RPB1*	TIM2 +F+I+G4	250	1247	880	899	899
4	*RPB2*	TIM +F+I+G4	285	820	519	559	559
5	ITS	GTR+F++I+G4	206	583	504	145	145

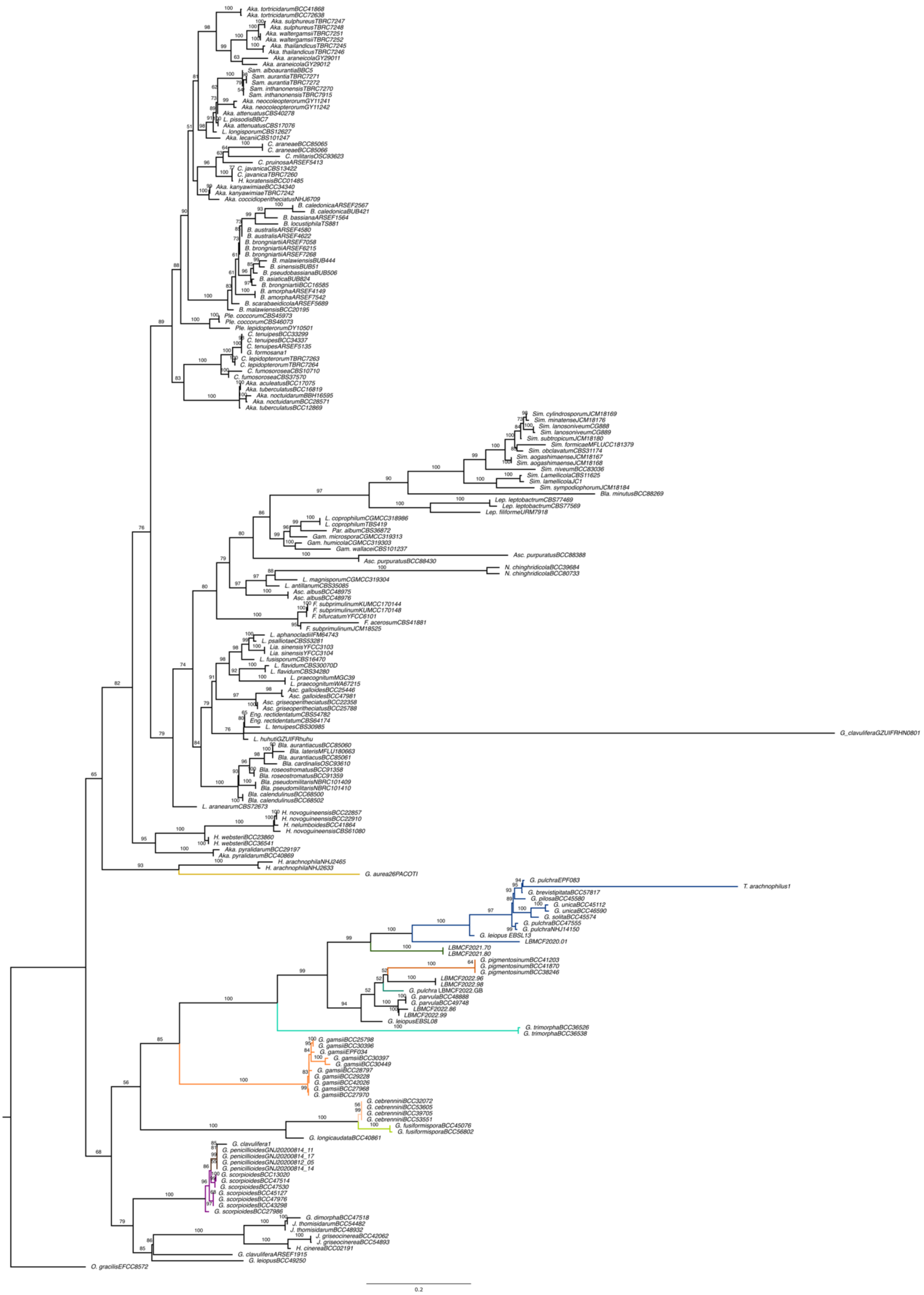

Figure S2: Phylogenetic analysis of the genus *Gibellula* using Maximum Likelihood with 1,000 bootstraps with the dataset of the Internal Transcribed Spacer (ITS) region.

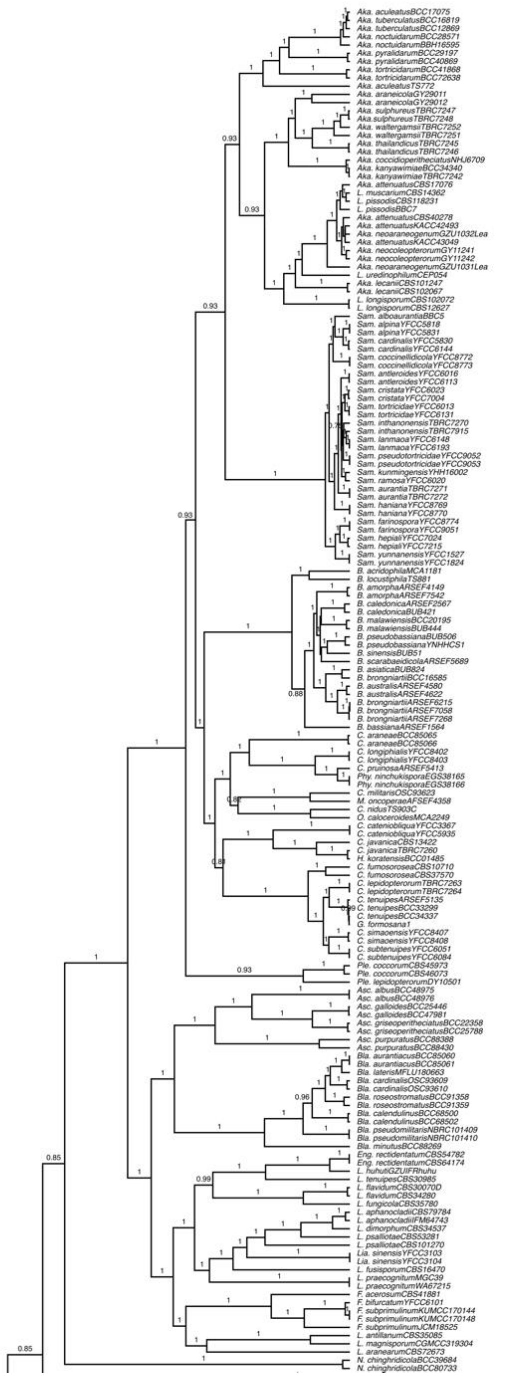
Aka. aculeatusBCC17075
Aka. tuberculatusBCC16819
Aka. tuberculatusBCC12869
Aka. noctuidarumBCC28571
Aka. noctuidarumBBH16595
Aka. pyralidarumBCC29197
Aka. pyralidarumBCC40869
Aka. tortricidarumBCC41868
Aka. tortricidarumBCC72638
Aka. aculeatusTS772
Aka. araneicolaGY29011
Aka. araneicolaGY29012
Aka. sulphureusTBRC7247
Aka.sulphureusTBRC7248
Aka. waltergamsiiTBRC7252
Aka. waltergamsiiTBRC7251
Aka. thailandicusTBRC7245
Aka. thailandicusTBRC7246
Aka. coccidioperitheciatusNHJ6709
Aka. kanyawimiaeBCC34340
Aka. kanyawimiaeTBRC7242
Aka. attenuatusCBS17076
L. muscariumCBS14362
L. pissodisCBS118231
L. pissodisBBC7
Aka. attenuatusCBS40278
Aka. attenuatusKACC42493
Aka. neoaraneogenumGZU1032Lea
Aka. attenuatusKACC43049
Aka. neocoleopterorumGY11241
Aka. neocoleopterorumGY11242
Aka. neoaraneogenumGZU1031Lea
L. uredinophilumCEP054
Aka. lecaniiCBS101247
Aka. lecaniiCBS102067
L. longisporumCBS102072
L. longisporumCBS12627
Sam. alboaurantiaBBC5
Sam. alpinaYFCC5818
Sam. alpinaYFCC5831
Sam. cardinalisYFCC5830
Sam. cardinalisYFCC6144
Sam. coccinellidicolaYFCC8772
Sam. coccinellidicolaYFCC8773
Sam. antleroidesYFCC6016
Sam. antleroidesYFCC6113
Sam. cristataYFCC6023
Sam. cristataYFCC7004
Sam. tortricidaeYFCC6013
Sam. tortricidaeYFCC6131
Sam. inthanonensisTBRC7270
Sam. inthanonensisTBRC7915
Sam. lanmaoaYFCC6148
Sam. lanmaoaYFCC6193
Sam. pseudotortricidaeYFCC9052
Sam. pseudotortricidaeYFCC9053
Sam. kunmingensisYHH16002
Sam. ramosaYFCC6020
Sam. aurantiaTBRC7271
Sam. aurantiaTBRC7272
Sam. hanianaYFCC8769
Sam. hanianaYFCC8770
Sam. farinosporaYFCC8774
Sam. farinosporaYFCC9051
Sam. hepialiYFCC7024
Sam. hepialiYFCC7215
Sam. yunnanensisYFCC1527
Sam. yunnanensisYFCC1824
B. acridophilaMCA1181
B. locustiphilaTS881
B. amorphaARSEF4149
B. amorphaARSEF7542
B. caledonicaARSEF2567
B. caledonicaBUB421
B. malawiensisBCC20195
B. malawiensisBUB444
B. pseudobassianaBUB506
B. pseudobassianaYNHHCS1
B. sinensisBUB51
B. scarabaeidicolaARSEF5689
B. asiaticaBUB824
B. brongniartiiBCC16585
B. australisARSEF4580
B. australisARSEF4622
B. brongniartiiARSEF6215
B. brongniartiiARSEF7058
B. brongniartiiARSEF7268
B. bassianaARSEF1564
C. araneaeBCC85065
C. araneaeBCC85066
C. longiphialisYFCC8402
C. longiphialisYFCC8403
C. pruinosaARSEF5413
Phy. ninchukisporaEGS38165
Phy. ninchukisporaEGS38166
C. militarisOSC93623
M. oncoperaeAFSEF4358
C. nidusTS903C
O. caloceroidesMCA2249
C. cateniobliquaYFCC3367
C. cateniobliquaYFCC5935
C. javanicaCBS13422
C. javanicaTBRC7260
H. koratensisBCC01485
C. fumosoroseaCBS10710
C. fumosoroseaCBS37570
C. lepidopterorumTBRC7263
C. lepidopterorumTBRC7264
C. tenuipesARSEF5135
C. tenuipesBCC33299
C. tenuipesBCC34337
G. formosana1
C. simaoensisYFCC8407
C. simaoensisYFCC8408
C. subtenuipesYFCC6051
C. subtenuipesYFCC6084
Ple. coccorumCBS45973
Ple. coccorumCBS46073
Ple. lepidopterorumDY10501
Asc. albusBCC48975
Asc. albusBCC48976
Asc. galloidesBCC25446
Asc. galloidesBCC47981
Asc. griseoperitheciatusBCC22358
Asc. griseoperitheciatusBCC25788
Asc. purpuratusBCC88388
Asc. purpuratusBCC88430
Bla. aurantiacusBCC85060
Bla. aurantiacusBCC85061
Bla. laterisMFLU180663
Bla. cardinalisOSC93609
Bla. cardinalisOSC93610
Bla. roseostromatusBCC91358
Bla. roseostromatusBCC91359
Bla. calendulinusBCC68500
Bla. calendulinusBCC68502
Bla. pseudomilitarisNBRC101409
Bla. pseudomilitarisNBRC101410
Bla. minutusBCC88269
Eng. rectidentatumCBS54782
Eng. rectidentatumCBS64174
L. huhutiGZUIFRhuhu
L. tenuipesCBS30985
L. flavidumCBS30070D
L. flavidumCBS34280
L. fungicolaCBS35780
L. aphanocladiiCBS79784
L. aphanocladiiIFM64743
L. dimorphumCBS34537
L. psalliotaeCBS53281
L. psalliotaeCBS101270
Lia. sinensisYFCC3103
Lia. sinensisYFCC3104
L. fusisporumCBS16470
L. praecognitumMGC39
L. praecognitumWA67215
F. acerosumCBS41881
F. bifurcatumYFCC6101
F. subprimulinumKUMCC170144
F. subprimulinumKUMCC170148
F. subprimulinumJCM18525
L. antillanumCBS35085
L. magnisporumCGMCC319304
L. aranearumCBS72673
N. chinghridicolaBCC39684
N. chinghridicolaBCC80733

4

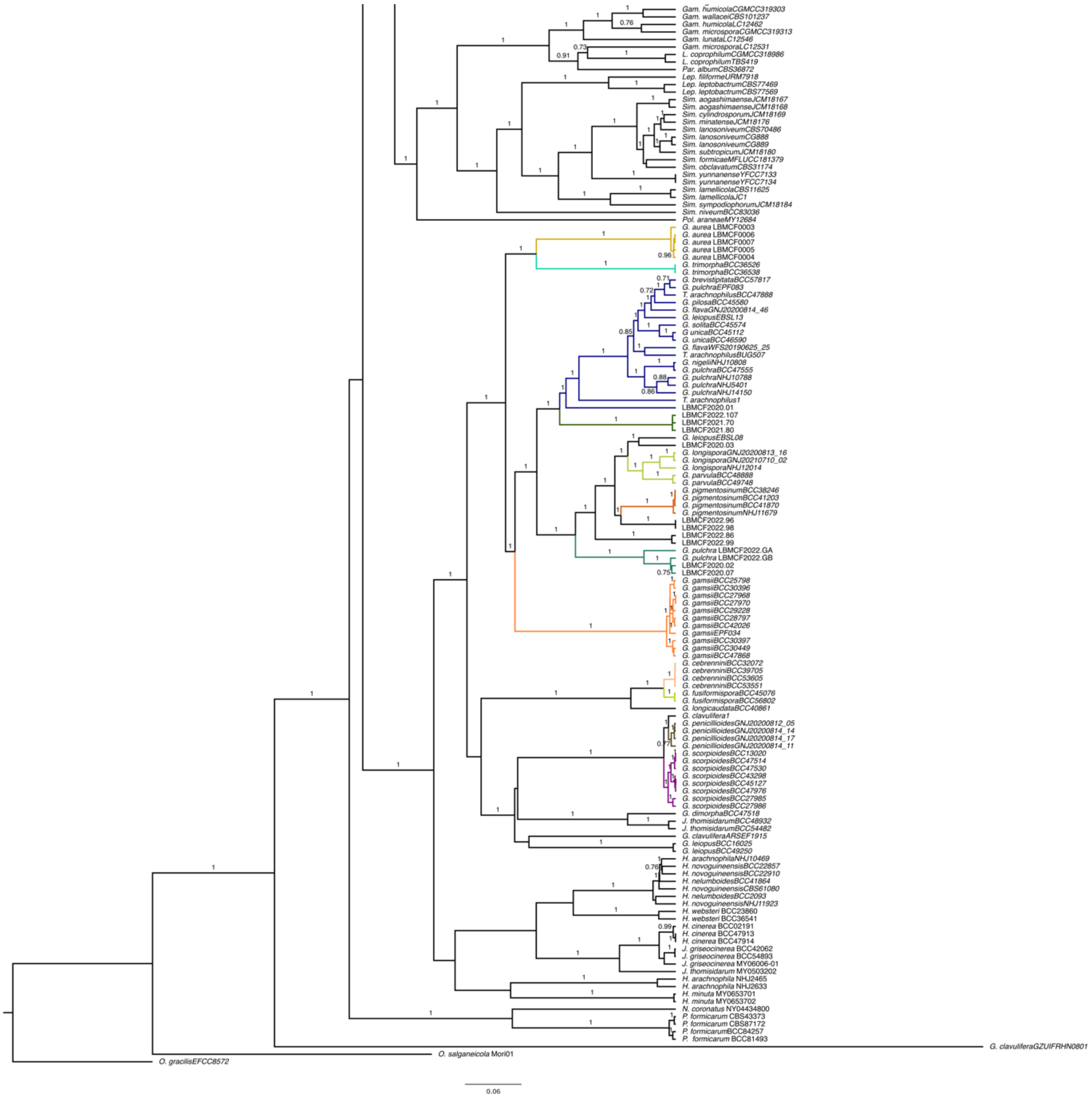

Figure S3: Phylogenetic analysis of the genus *Gibellula* using Bayesian Inference with 30 million generations with the concatenated dataset of the six genomic regions: small (SSU) and large (LSU) nuclear ribosomal subunits, Internal Transcribed Spacer (ITS), translation elongation factor 1-α (TEF), RNA polymerase II largest subunit (RPB1), and RNA polymerase II second largest subunit (RPB2).

References

1. Page, M.J.; McKenzie, J.E.; Bossuyt, P.M.; Boutron, I.; Hoffmann, T.C.; Mulrow, C.D.; Shamseer, L.; Tetzlaff, J.M.; Akl, E.A.; Brennan, S.E.; et al. The PRISMA 2020 Statement: An Updated Guideline for Reporting Systematic Reviews. *BMJ* **2021**, 372:71, doi: https://doi.org/10.1136/bmj.n71.
2. White, T.J.; Bruns, T.; Lee, S.; Taylor, J. Amplification and Direct Sequencing of Fungal Ribosomal RNA Genes for Phylogenetics. In *PCR Protocols: A Guide to*

Methods and Application; Innis, M.A., Gelfand, D.H., Sninsky, J.J., White, T.J., Eds.; Elsevier, Academic Press: New York, 1990; pp. 315–322.
3. Vilgalys, R.; Sun, B.L. Ancient and Recent Patterns of Geographic Speciation in the Oyster Mushroom *Pleurotus* Revealed by Phylogenetic Analysis of Ribosomal DNA Sequences. *Proc. Natl. Acad. Sci.* **1994**, *91*, 4599–4603, doi:10.1073/pnas.91.10.4599.
4. Vilgalys, R.; Hester, M. Rapid Genetic Identification and Mapping of Enzymatically Amplified Ribosomal DNA from Several *Cryptococcus* Species. *J. Bacteriol.* **1990**, *172*, 4238–4246, doi:10.1128/jb.172.8.4238-4246.1990.
5. Castlebury, L.A.; Rossman, A.Y.; Sung, G.-H.; Hyten, A.S.; Spatafora, J.W. Multigene Phylogeny Reveals New Lineage for *Stachybotrys Chartarum*, the Indoor Air Fungus. *Mycological Research* **2004**, *108*, 864–872, doi:10.1017/S0953756204000607.
6. Murata, N.; Aoki, T.; Kusaba, M.; Tosa, Y.; Chuma, I. Various Species of Pyricularia Constitute a Robust Clade Distinct from Magnaporthe Salvinii and Its Relatives in Magnaporthaceae. *J Gen Plant Pathol* **2014**, *80*, 66–72, doi:10.1007/s10327-013-0477-z.
7. Liu, Y.J.; Whelen, S.; Hall, B.D. Phylogenetic Relationships among Ascomycetes: Evidence from an RNA Polymerse II Subunit. *Molecular Biology and Evolution* **1999**, *16*, 1799–1808, doi:10.1093/oxfordjournals.molbev.a026092.
8. Araújo, J.P.M.; Evans, H.C.; Kepler, R.; Hughes, D.P. Zombie-Ant Fungi across Continents: 15 New Species and New Combinations within *Ophiocordyceps*. I. Myrmecophilous Hirsutelloid Species. *Studies in Mycology* **2018**, *90*, 119–160, doi:10.1016/j.simyco.2017.12.002.
9. Kalyaanamoorthy, S.; Minh, B.Q.; Wong, T.K.F.; von Haeseler, A.; Jermiin, L.S. ModelFinder: Fast Model Selection for Accurate Phylogenetic Estimates. *Nat Methods* **2017**, *14*, 587–589, doi:10.1038/nmeth.4285.
10. Chernomor, O.; von Haeseler, A.; Minh, B.Q. Terrace Aware Data Structure for Phylogenomic Inference from Supermatrices. *Syst Biol* **2016**, *65*, 997–1008, doi:10.1093/sysbio/syw037.
